# A straightforward approach for bioorthogonal labeling of proteins and organelles in live mammalian cells, using a short peptide tag

**DOI:** 10.1186/s12915-019-0708-7

**Published:** 2020-01-14

**Authors:** Inbar Segal, Dikla Nachmias, Andres Konig, Ariel Alon, Eyal Arbely, Natalie Elia

**Affiliations:** 1 Department of Life Sciences, Ben-Gurion University of the Negev, 84105 Beer Sheva, Israel; 2 National Institute for Biotechnology in the Negev (NIBN), Ben-Gurion University of the Negev, 84105 Beer Sheva, Israel; 3 Department of Chemistry, Ben-Gurion University of the Negev, 84105 Beer Sheva, Israel

## Abstract

**Background:**

In the high-resolution microscopy era, genetic code expansion (GCE)-based bioorthogonal labeling offers an elegant way for direct labeling of proteins in live cells with fluorescent dyes. This labeling approach is currently not broadly used in live-cell applications, partly because it needs to be adjusted to the specific protein under study.

**Results:**

We present a generic, 14-residue long, N-terminal tag for GCE-based labeling of proteins in live mammalian cells. Using this tag, we generated a library of GCE-based organelle markers, demonstrating the applicability of the tag for labeling a plethora of proteins and organelles. Finally, we show that the HA epitope, used as a backbone in our tag, may be substituted with other epitopes and, in some cases, can be completely removed, reducing the tag length to 5 residues.

**Conclusions:**

The GCE-tag presented here offers a powerful, easy-to-implement tool for live-cell labeling of cellular proteins with small and bright probes.

## Background

Tracking the dynamics of proteins and organelles in live cells is key to understanding their functions. For this, fluorescent protein (e.g., GFP) or self-labeling protein (e.g., Halo-Tag) tags are routinely attached to proteins in cells [1]. While these tags are vigorous and easy to implement, they are large and bulky (e.g., GFP,  27 kDa; Halo-tag, 33 kDa), such that their attachment could affect the dynamics and function of the protein under study. Using genetic code expansion (GCE) and bioorthogonal chemistry, it is now possible to attach fluorescent dyes (Fl-dyes) to specific protein residues, thereby allowing direct labeling of proteins in live cells with Fl-dyes [1–3]. Indeed, this approach has been applied, in recent years, for fluorescent labeling of extra- and intracellular proteins [4–10].

In GCE-based labeling, a non-canonical amino acid (ncAA) carrying a functional group is incorporated into the sequence of a protein in response to an in-frame amber stop codon (TAG), via an orthogonal tRNA/tRNA-synthetase pair (reviewed in [11, 12]). Labeling is then carried out by a rapid and specific bioorthogonal reaction between the functional group and the Fl-dye [2, 4, 8, 9, 13, 14]. Successful labeling hence relies on the exogenous expression of an orthogonal tRNA/tRNA-synthetase pair and a protein of interest (bearing a ncAA) at sufficient levels to allow effective labeling.

The ncAA (and consequently the Fl-dye) can, in theory, be incorporated anywhere in the protein sequence. In practice, however, finding a suitable labeling site can be laborious and time-consuming for several reasons. First, prior knowledge or functional assays are necessary to ensure that the insertion of the ncAA at a specific position does not affect protein structure and function [4–7, 10]. Second, the efficiency of ncAA incorporation varies at different locations in the protein with no guidelines for the preferred sequence context having been reported [3–7, 15]. Notably, low efficiency of ncAA incorporation does not only lead to ineffective labeling but also to the translation of a truncated version of the protein (resulting from the insertion of a premature stop codon), which can be toxic to cells [5, 6, 16, 17]. Third, the ncAA should be incorporated in a position that will allow the functional group to be accessible to the solvent to enable efficient bioorthogonal conjugation with the Fl-dye. All these requirements are protein specific, such that any attempt at labeling via this approach begins with a screen for suitable incorporation sites [2–4, 6, 7]. Consequently, despite its great potential, GCE-based labeling is presently not widely used in mammalian live-cell imaging studies [5].

**Fig. 1:**
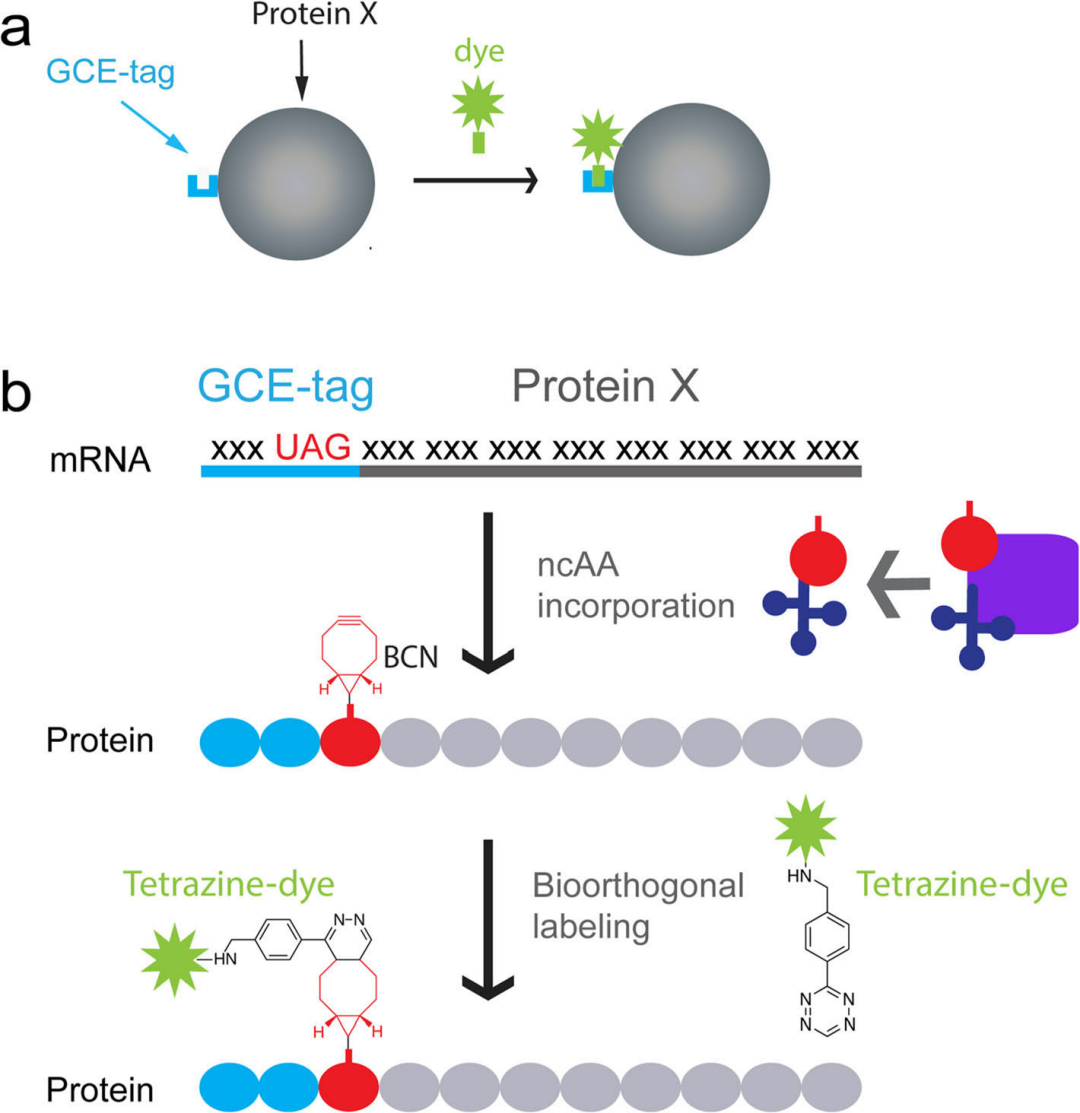
Using a GCE-tag for fluorescence labeling of proteins via genetic code expansion (GCE) and bioorthogonal chemistry. **a** Schematic representation of the labeling approach. A GCE-tag (blue) is attached to the N-terminal of the protein (gray). Binding of the Fl-dye (green) to the GCE-tag leads to labeling of the protein. **b** Graphical illustration of the experimental design. The stop codon, UAG, and nucleotides encoding a short polypeptide tag are added upstream to the coding sequence of a protein. During ribosomal translation, the ncAA BCN-Lys (red) is incorporated into the protein in response to the in-frame UAG codon using a specific, orthogonal pair of tRNA (dark blue)/tRNA-Synth (purple). Finally, a tetrazine-conjugated Fl-dye (green) is covalently attached to BCN-Lys via a bioorthogonal reaction. As a result, the protein is directly labeled with Fl-dye via a small polypeptide tag.

To bypass the screening steps currently associated with GCE-based labeling and expand the use of the technique, we set out to design a minimal peptide tag that encodes the incorporation of a ncAA through GCE, for bioorthogonal labeling of proteins in live cells; we term this tag a GCE-tag (Fig. 1a). Using the HA epitope as a backbone, we optimized a 14-residue long N-terminal tag and demonstrated its applicability for live-cell labeling of proteins and organelle markers in mammalian cells. With this tag in hand, cellular proteins can now be labeled via GCE and bioorthogonal chemistry for live-cell imaging applications with no screening steps required.

## Results

Proteins can potentially be tagged at their N- or C-terminal. For GCE-based labeling, we chose to design an N-terminal tag in order to avoid protein truncations resulting from inefficient incorporation of the ncAA [5, 6, 16, 17]. On the basis of our previous work, potential tags were cloned into a single expression vector, designed to encode the incorporation of the ncAA bicyclo-nonyne Lysine (BCN-Lys) that bioorthogonally reacts with tetrazine-conjugated Fl-dyes (Fig. 1b and Additional file 1: Figure S1a) [6, 18, 19]. Labeling potency was initially assessed using α-tubulin as a benchmark and evaluating microtubule (MT) labeling in live mammalian cells in the presence of Silicon-Rhodamine-Tetrazine (SiR-Tet) [6, 20]. MT labeling obtained upon site-specific incorporation of BCN-Lys in α-tubulin at position 45 (α-tubulin^45TAG^) was used as a reference, given our earlier demonstration of the efficacy of MT labeling at this site [6].

**Fig. 2:**
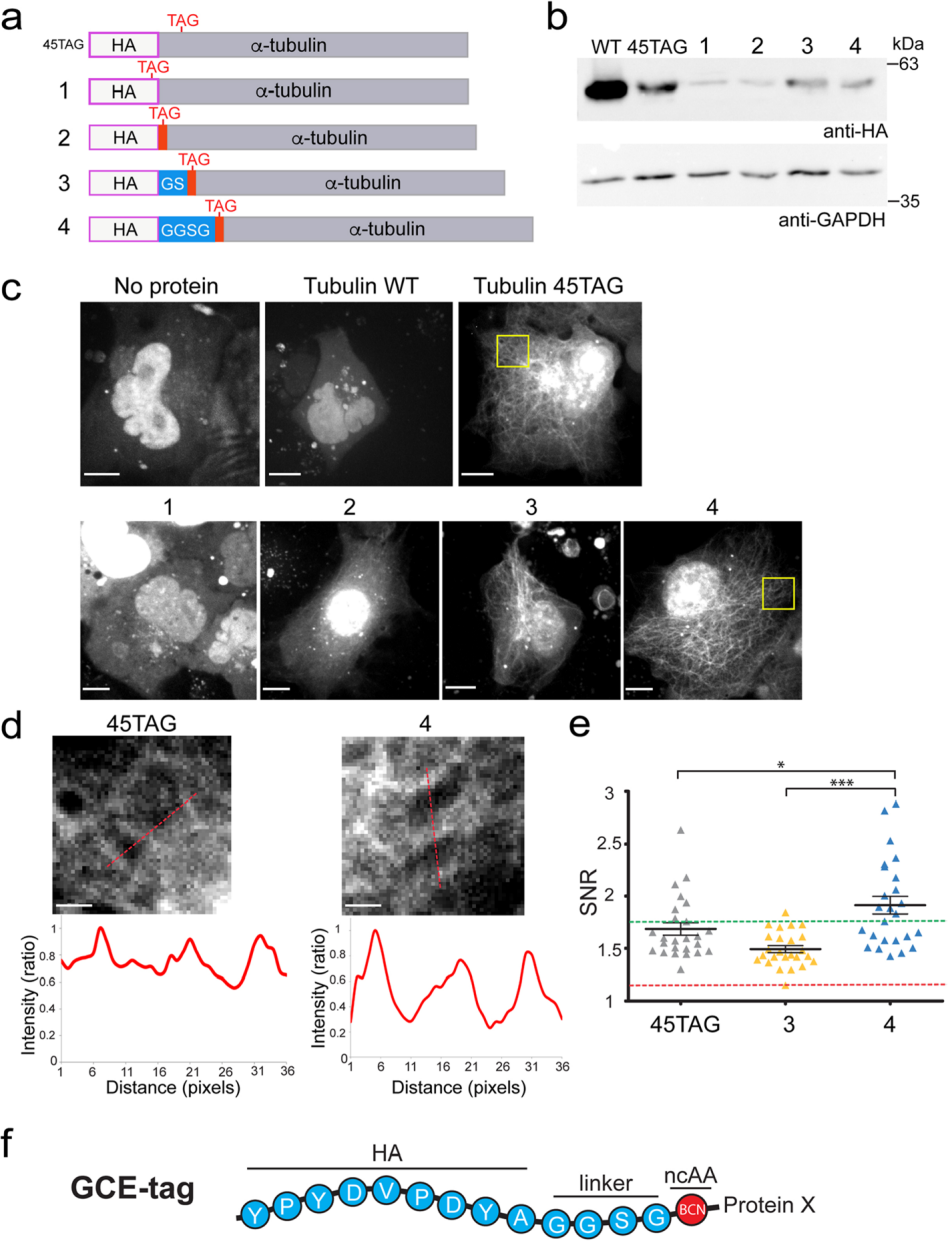
Optimizing a minimal tag for genetic code expansion (GCE)-based fluorescence labeling using α-tubulin. **a** Schematic view of the tags used and tested in **b**–**e**. Complete sequences of the tags are shown in Additional file 1: Figure S1c-f. **b**–**e** GCE-tags (specified in a) were sub-cloned to the N-terminal of α-tubulin using the pBUD-Pyl-RS-tub plasmid (Additional file 1: Figure S1a). HEK293T (**b**) or COS7 (**c**–**e**) cells were transfected with pBUD-Pyl-RS-tub plasmids carrying different tags, a WT version of α-tubulin, or without a target protein, and incubated for 48 h in the presence of the ncAA BCN-Lys. Cells were then subjected to western blot analysis (**b**) or labeled with SiR-Tet for 1 h and imaged live (**c**–**e**). Images in **c** are maximum intensity projections of 3D z-stacks obtained from representative cells. **d** Zoomed-in images of the regions marked in yellow squares in **c**. Intensity values along the red lines drawn in the images are plotted below, demonstrating the improvement in SNR. **e** SNR values measured in cells expressing α-tubulin with BCN-Lys incorporated either in position G45 or in the N-terminal via tags 3 or 4. The averaged SNR measured in cells expressing WT tubulin was 1.2 (red dashed line). The averaged SNR previously measured using GFP was 1.75 (green dashed line). *n* = 25. Results presented in **b**–**e** were obtained in at least 3 independent experiments. **f** Schematic view of the optimized GCE-tag used for further labeling. Scale bars: **c** 10 μm, **d** 2 μm

The most minimal N-terminal GCE-tag should include a TAG codon that will encode the incorporation of BCN-Lys. Unfortunately, no specific labeling was obtained upon expressing α-tubulin that carries a TAG codon at its N-terminal (directly before the first residue) in the presence of SiR-Tet, using live-cell imaging or in-gel fluorescence (Additional file 1: Figure S1b and S2). This indicates that the sole addition of a TAG codon upstream of a protein coding sequence is not sufficient for labeling, perhaps due to the failure of the ribosome to incorporate the ncAA at the very N-terminal of a protein. Potential N-terminal tags, bearing different lengths, were therefore designed based on the commonly used nine amino-acid (AA)-long hemagglutinin (HA) epitope as a backbone [21] (Fig. 2a and Additional file 1: Figure S1c-f). The TAG codon was either introduced within the HA sequence (replacing the last codon in the epitope) (1), immediately after the HA sequence (2), or after a short commonly used flexible linker comprising either glycine-serine (GS; 3) or glycine-glycine-serine-glycine (GGSG; 4). Low but noticeable levels of α-tubulin were observed using any of these tags, indicating that N-terminally tagged α-tubulin was expressed in cells (Fig. 2b). In live-cell labeling experiments with SiR-Tet, little to no MT decoration was obtained using α-tubulin tagged with probes 1 or 2 (Fig. 2c, 1–2), while clear and specific labeling was observed using tags 3 and 4 (Fig. 2c, 3–4), indicating that probes 3 and 4 can potentially serve as GCE-tags for α-tubulin.

To evaluate labeling efficiencies obtained using potential GCE-tags, signal-to-noise ratios (SNRs) were measured in labeled cells based on line intensity profiles, as illustrated in Fig. 2d. SNR values measured in cells expressing any of the α-tubulin versions were significantly higher than the average background levels measured in cells expressing a wild type (WT) version of α-tubulin that is unable to incorporate the ncAA, verifying that labeling is specific (Fig. 2e). Cells expressing α-tubulin tagged with probe 4 exhibited significantly higher SNR compared to probe 3, indicating that HA-GGSG-ncAA is preferable over HA-GS-ncAA for MT labeling. Notably, despite the relatively low expression levels obtained using probe 4, the average SNR values obtained in cells expressing α-tubulin tagged with probe 4 were higher than those obtained in cells expressing α-tubulin^45TAG^ or in cells expressing α-tubulin-GFP (Fig. 2d, e) [6]. Improved SNRs under lower α-tubulin expression levels can result from reducing the fraction of cytosolic (non-polymerized)-labeled tubulin in cells and is preferential for cell physiology. We therefore concluded that HA-GGSG-ncAA-tagged α-tubulin is superior to site-specific ncAA-incorporated α-tubulin for MT labeling. Based on these results we defined the N′ HA-GGSG-ncAA as the minimal tag for GCE-based bioorthogonal labeling of proteins in live mammalian cells (Fig. 2f).

**Fig. 3:**
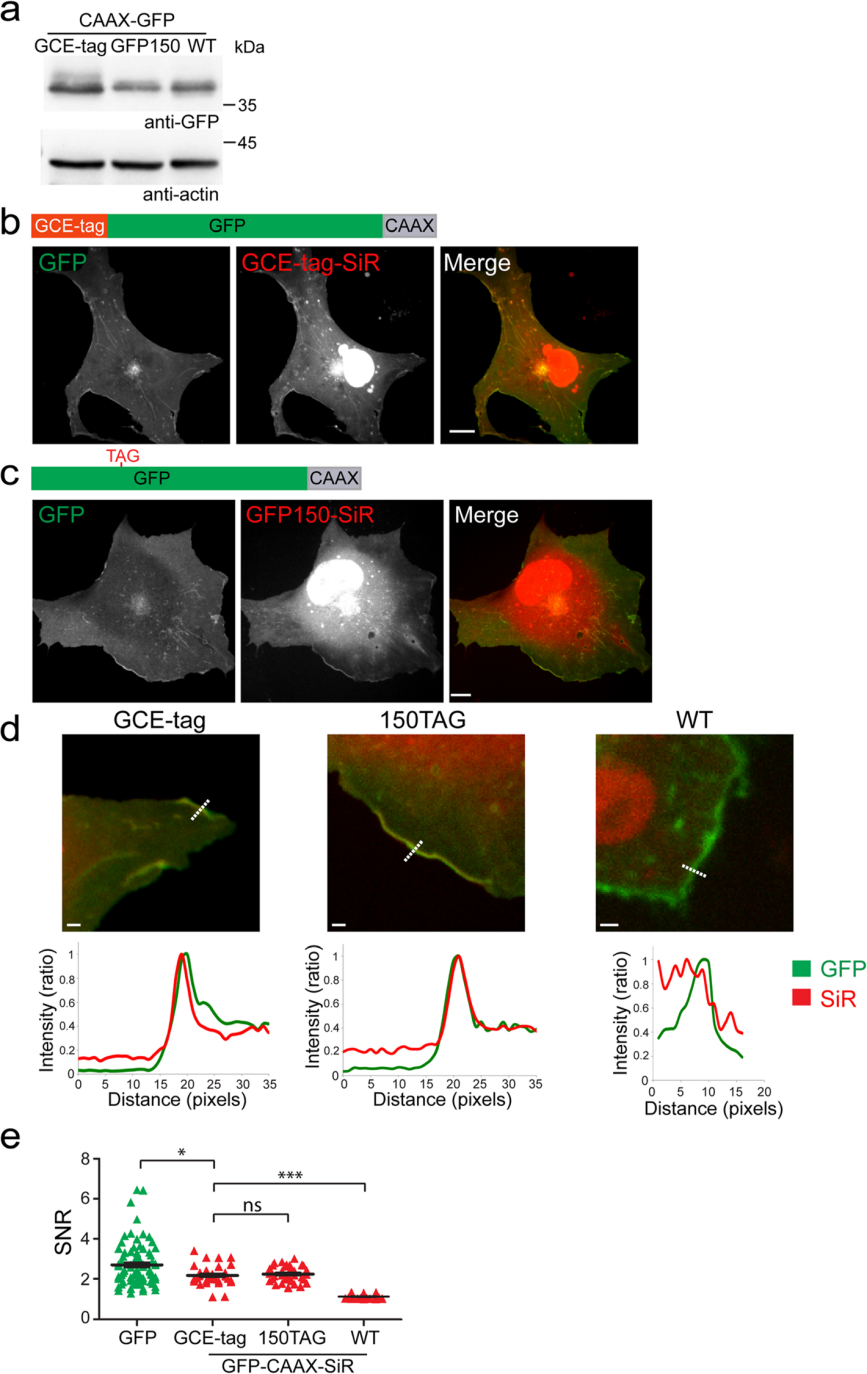
Plasma membrane (PM) labeling obtained via the minimal GCE-tag is comparable to GFP and site-specific labeling. HEK293T (**a**) or COS7 (**b**–**e**) cells were transfected with pBUD-Pyl-RS plasmids carrying GCE-tag-GFP-CAAX, GFP-CAAX mutated at position 150 to TAG or a WT version of GFP-CAAX, and incubated for 48 h in the presence of BCN-Lys. Cells were then subjected to western blot analysis (**a**) or labeled with SiR-Tet for 1 h and imaged live (**b**–**e**). Images represent single confocal slices of representative cells. **d** Line intensity profiles obtained in cells expressing the indicated constructs and labeled with SiR-Tet. Upper panel: zoomed-in images of confocal slices. Bottom panel: intensity values along the dashed line drawn in the images. **e** SNR values measured for GFP and SiR in cells expressing the different versions of GFP-CAAX. *n* = 30. Results were obtained in at least 3 independent experiments. Scale bars: **b**, **c** 10 μm, **d** 2 μm

**Fig. 4:**
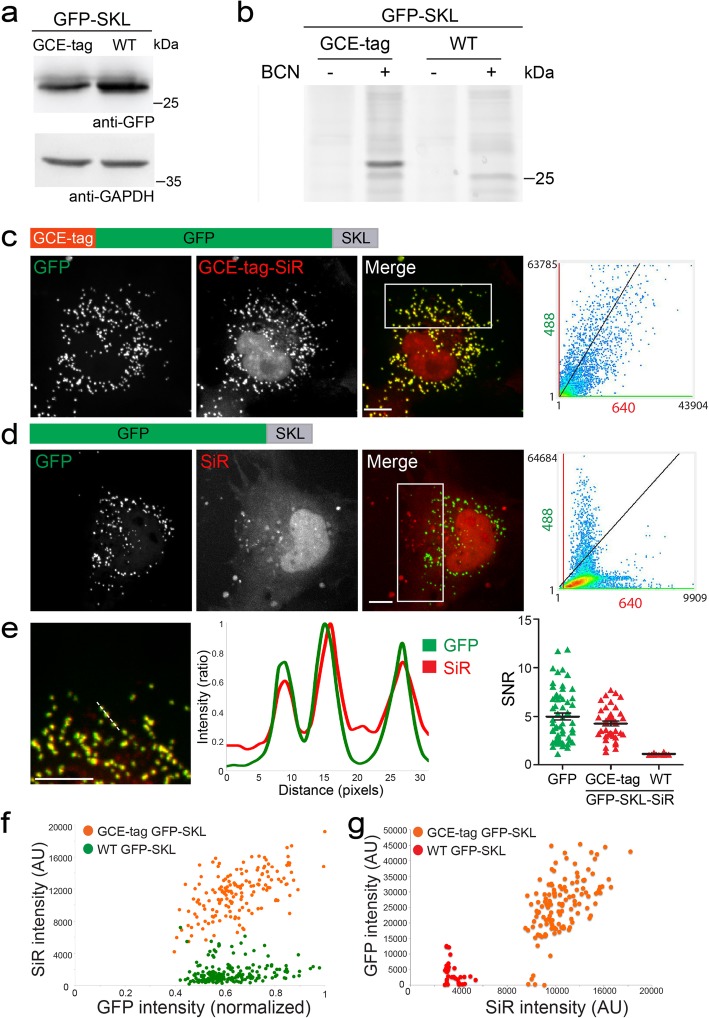
Demonstrating labeling specificity and efficiency in peroxisomes labeled with GCE-tagged GFP-SKL. HEK293T (**a**, **b**) or COS7 (**c**–**f**) cells were transfected with pBUD-Pyl-RS plasmids carrying the indicated GFP-SKL constructs and incubated for 48 h in the presence of the ncAA BCN-Lys (except for lanes 1 and 3 in **b**). Cells were then subjected to western blot analysis (**a**) or labeled with SiR-Tet for 1 h and subjected to in-gel fluorescence (**b**) or imaged live (**c**–**g**). **b** Cells were lysed, and equal total protein amounts were loaded on an SDS-PAGE. Results were recorded using SiR fluorescence. A specific band obtaining a similar size to that obtained in western blot was seen only in cells expressing GCE-tag-GFP-SKL and incubated with BCN-Lys, indicating specific labeling. **c**, **d** Maximum intensity projections of representative cells. Left to right: fluorescence obtained in each channel, merged image and co-localization analysis between intensity values of the 640 (SiR) and 488 (GFP) on a large subset of the cell that excludes the nucleus (white rectangle in merged image): Pearson’s correlation values: **c** 0.86, **d** 0.07. **e** Left to right: a zoomed-in image of the cell in **c**, a line intensity profile measured for the dashed line in the zoomed-in image, and a summary of SNR values measured for GFP and SiR in cells expressing the different versions of GFP-SKL. *n* = 40. **f**, **g** Cells expressing the indicated constructs were labeled with SiR-Tet and imaged using similar imaging parameters (exposure time and laser intensity). **f** GFP-positive peroxisomes were segmented based on intensity. For each peroxisome, the mean intensity in the GFP channel was plotted against the mean intensity in the SiR channel. ~ 95% of GFP-labeled peroxisomes exhibited SiR intensity levels that are above background. **g** SiR-positive puncta were segmented based on intensity. For each punctum, the mean intensity in the SiR channel was plotted against the mean intensity in the GFP channel. ~ 5% of SiR-positive puncta were GFP negative. Mean intensity levels measured for SiR puncta in cells expressing GCE-tag-GFP-SKL were at least twofolds higher than the levels measured for SiR puncta segmented in control cells expressing WT GFP-SKL, indicating specific labeling. Results were obtained in at least 3 independent experiments. Scale-bars 10 μm

To examine the applicability and specificity of the GCE-tag (HA-GGSG-ncAA, Fig. 2f and Additional file 1: Figure S1f) for labeling different cellular compartments, we first applied it to cellular markers that are conjugated to GFP, i.e., the plasma membrane (PM) marker GFP-CAAX and the peroxisomal marker GFP-SKL (Figs. 3 and 4). GCE-tagged GFP-CAAX was expressed in cells at higher levels than GFP-CAAX that carries a TAG codon at the optimized position in GFP (GFP^150TAG^-CAAX, Fig. 3a) [19]. Consistently, PM decoration was observed in cells expressing either GCE-tagged GFP-CAAX or GFP^150TAG^-CAAX and labeled with SiR-Tet in both the GFP and SiR channels with relatively similar SNR values (Fig. 3b, c). SNR values measured for PM labeling using the SiR channel for GCE-tagged GFP-CAAX or GFP^150TAG^CAAX were similar and significantly higher than the levels measured in cells expressing GFP-CAAX that is unable to incorporate the ncAA (WT GFP-CAAX), indicating that labeling is specific and is not compromised by the GCE-tag (Fig. 3d, e).

GCE-tagged GFP-SKL was expressed in HEK293 cells, and a specific band at a similar size was observed in an in-gel fluorescence assay upon labeling the cells with SiR-Tet, in the presence of BCN-Lys (Fig. 4a, b). Notably, while WT GFP-SKL was expressed at higher levels compared to GCE-tagged GFP-SKL, no specific labeling was observed for WT GFP-SKL using in-gel fluorescence, strongly indicating that labeling is specifically induced by binding of the Fl-dye to the ncAA in GCE-tagged GFP-SKL. In live cells, remarkable co-localization of GFP and SiR in small puncta, was observed throughout the cell (except in the nucleus) at similar SNRs (Fig. 4c, Pearson’s correlation = 0.86). This was not the case in cells expressing WT GFP-SKL and labeled with SiR, which exhibited an anti-correlation between GFP and SiR puncta (Fig. 4d, e, Pearson’s correlation = 0.07). In these cells, a small population of SiR puncta was observed but these puncta did not co-localize with the GFP puncta observed throughout the cell. These results further indicate that the co-localization observed in cells expressing GCE-tagged GFP-SKL resulted from specific SiR labeling of the protein.

The organization of the fluorescence signal into discrete puncta in SKL labeled cells allowed us to estimate relative labeling yields by segmenting the population of puncta in one channel and quantifying their intensities in the other channel (Fig. 4f, g). GFP puncta segmented from cells expressing WT GFP-SKL and labeled with SiR-Tet showed low intensity SiR levels that did not scale with GFP intensity levels (Fig. 4f). We therefore reasoned that the SiR levels obtained in these cells represent background SiR fluorescence. In cells expressing GCE-tagged GFP-SKL, the majority of segmented GFP puncta (~ 95%) had higher SiR intensity levels than those measured in cells expressing WT GFP-SKL. Moreover, intensity levels in the SiR channel scaled with GFP intensities, indicating that SiR fluorescence can be used for quantifying the relative levels of GFP-SKL in single peroxisomes. Segmenting the SiR-positive puncta in cells expressing GCE-tagged GFP-SKL globally resulted in similar behavior, with a small population (less than 5%) of SiR puncta that appeared GFP negative (Fig. 4g). In cells expressing WT GFP-SKL, a small population of SiR puncta was segmented. These puncta had low intensity levels in both GFP and SiR channels compared to SiR puncta segmented in GCE-tagged GFP-SKL expressing cells, indicating that they represent noise. These structures can be easily filtered out based on intensity, using image processing. Taken together, these data indicate that GCE-tag SiR-based labeling has ~ 95% yield compared to GFP and that it can be used for quantitative live-cell imaging of peroxisomes.

**Fig. 5:**
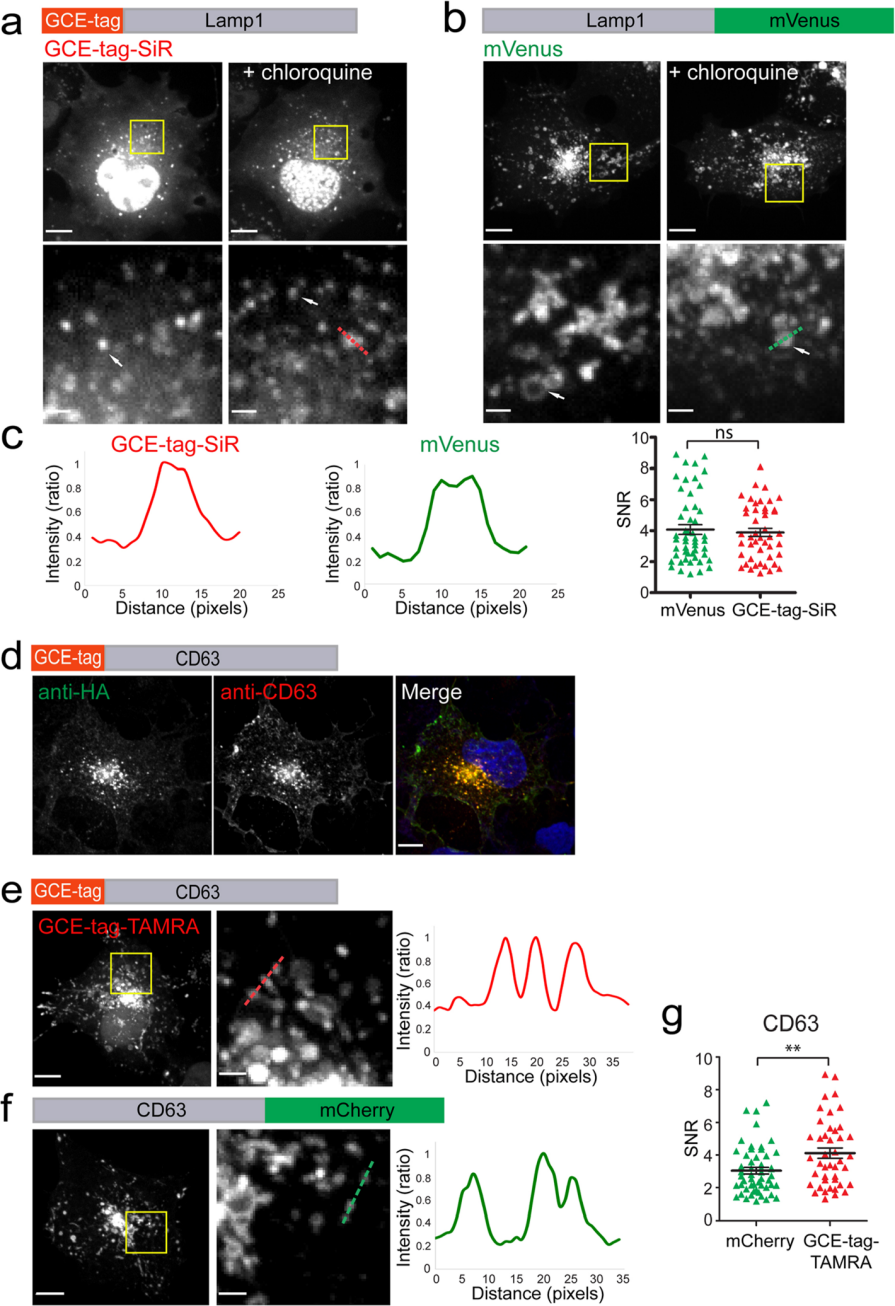
Efficient lysosome labeling is obtained using SiR-Tet while MVB labeling requires TAMRA-Tet. **a**–**c** Cells expressing GCE-tag-Lamp1 labeled with SiR-Tet (**a**) or Lamp1-mVenus (**b**). Left panels: no treatment. Right panels: treatment with the lysosome inhibitor chloroquine (3 h, 120 μM). Zoomed in images of a subset of the cells (correspond to squares in upper panels) are shown in bottom panels. Arrows indicate filled versus hollow lysosome labeling. **c** Left to right: line intensity profiles measured for the red dashed line in **a**, the green dashed line in **b**, and a summary of SNR values measured in cells expressing the indicated plasmids (*n* = 50). **d** COS7 cells expressing the MVB marker CD63 conjugated to the GCE-tag were fixed and stained with anti-HA and anti-CD63 antibodies. **e**, **f** MVB labeling in cells expressing GCE-tag-CD63 labeled with TAMRA-Tet (**e**) or CD63-mCherry (**f**). **g** SNR values measured in cells expressing the indicated plasmids. *n* = 50. Results presented in each panel were obtained in at least 3 independent experiments. Scale-bars, 10 μm; zoomed-in images, 2 μm

Next, we tested the robustness of the tag by applying it to diverse cellular organelles. For that, we used Lamp1 as a lysosomal marker, CD63 as a marker of multivesicular bodies (MVBs), ER^cb5^TM as an endoplasmic reticulum (ER) marker, Exo70 as an exosomal marker, and Mito^cb5^TM(mito) or mito-DsRed as mitochondrial markers (for sequences see Additional file 1: Table S1). Nuclear labeling was avoided due to the known non-specific labeling of the nucleus observed with GCE-based labeling (Figs. 2c; 3b, c; and 4c, d) [4, 6, 16]. Effective lysosome labeling was obtained using GCE-tagged Lamp1 in the presence of SiR-Tet (Fig. 5a, b). Overall, labeling was comparable to that obtained with Lamp1-mVenus. Lysosomes labeled via either of the tags (i.e., GCE-tag or mVenus) exhibited a similar response to chloroquine treatment and had similar SNR levels (Fig. 5a–c) [22]. We noticed that while lysosome labeling using Lamp1-mVenus highlighted both filled and hollow structures, only filled structures were observed in cells labeled with GCE-tag-SiR-Lamp1. This difference may result from the position of the tag; the mVenus tag faces the cytosol, while the GCE-tag faces the lysosomal lumen. Labeling lysosomal lumen proteins using conventional fluorescent proteins is challenging due to the pH sensitivity of the latter [23]. Our data suggest that GCE-tag-based labeling is compatible with labeling acidic cellular structures.

**Fig. 6:**
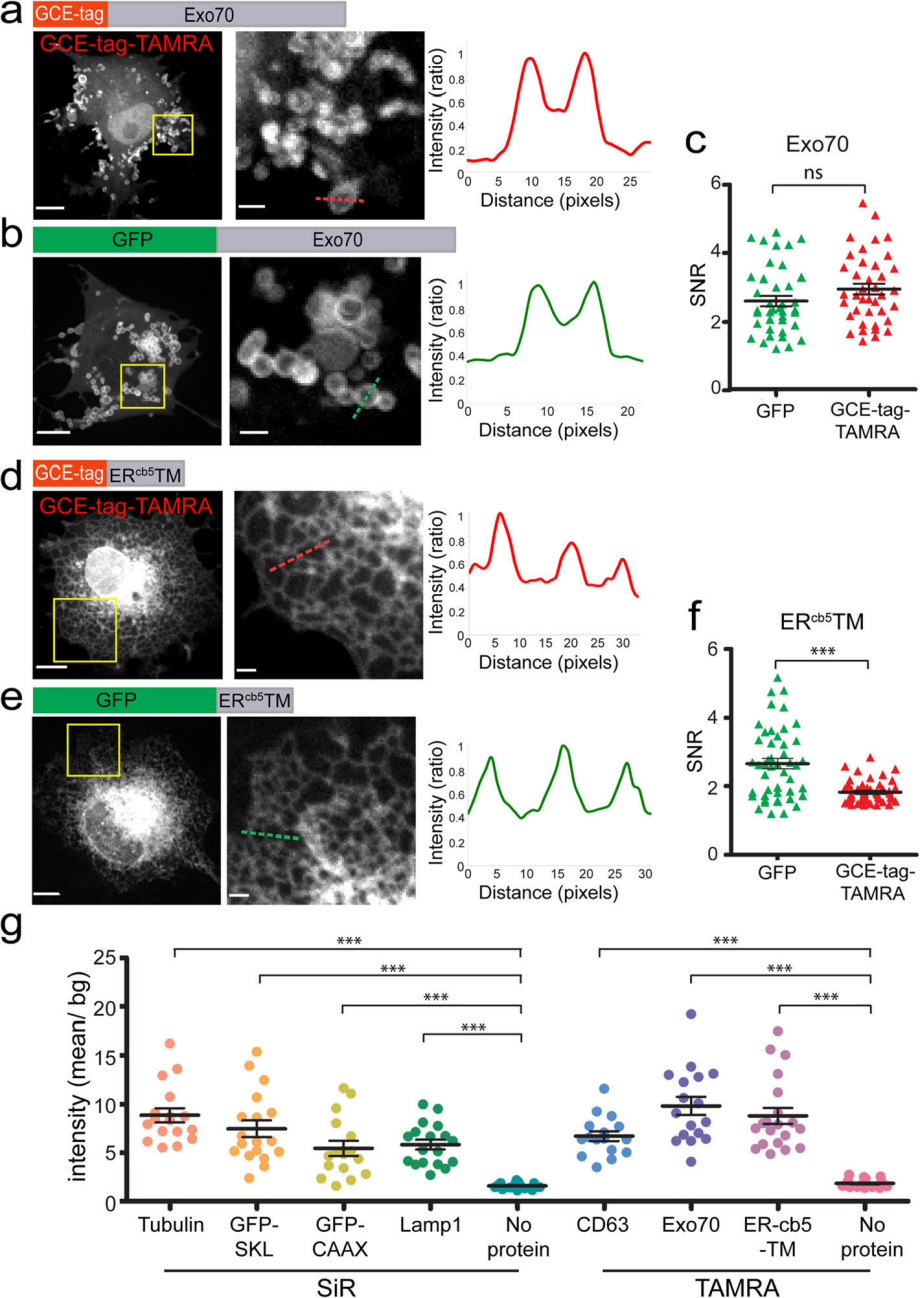
Exosomes and ER exhibit specific cellular labeling via the minimal GCE-tag in the presence of TAMRA-Tet. COS7 cells were transfected with the indicated plasmids, labeled when indicated, and imaged live 48 h later. **a**, **b**, **d**, **e** Left to right: maximum projection images, zoomed-in images of a subset of the cells (correspond to squares in left panels), line intensity profiles measured for the dashed lines in middle panels. **c**, **f** SNR values measured in cells expressing the indicated plasmids (*n* Exo70 = 40, ER^cb5^TM = 45). **g** Cells were transfected with the indicated the pBUD-Pyl-RS plasmids and labeled with SiR-Tet or TAMRA-Tet, as indicated. For each condition, mean intensity levels were measured for a region of interest inside the cell and were divided by the mean background intensity measured outside the cell. pBUD-Pyl-RS plasmids that do not encode for a protein of interest were used as negative controls (no protein). *n* = 15. Results presented in each panel were obtained in at least 3 independent experiments. Scale-bars, 10 μm; zoomed-in images, 2 μm

Initial attempts at labeling MVBs, ER, mitochondria, and exosomes using the GCE-tag and SiR-Tet resulted in no specific staining (Additional file 1: Figure S3). Yet, in immunostaining experiments using anti-CD63 antibodies, GCE-tagged CD63 co-localized with endogenous CD63 (Fig. 5d). We thus reasoned that GCE-tagged CD63 was properly expressed and targeted in cells, yet failed to bind the Fl-dye via the bioorthogonal reaction. Consistent with this notion, substituting TAMRA-Tet for SiR-Tet resulted in specific labeling of MVBs, ER, and exosomes using GCE-tagged CD63, ER^cb5^TM, and Exo70, respectively (Figs. 5e–g and 6). Mitochondrial labeling was not obtained using either SiR- or TAMRA-Tet together with the GCE-tag-Mito^cb5^TM or mito-DsRed probes (Additional file 1: Figure S4). Based on immunofluorescence experiments, it appears that while Mito^cb5^TM failed to localize to mitochondria, GCE-tagged mito-DsRed localized properly but failed to bind the tetrazine-conjugated dye (Additional file 1: Figure S4). Expression patterns and SNR values obtained with the GCE-tagged organelle markers that were successfully labeled (i.e., for MVBs, ER, and exosomes) were overall comparable to those obtained using conventional Fl-protein markers with better SNRs obtained for GCE-tagged MVBs and lower SNRs obtained for GCE-tagged ER (Figs. 5e–g and 6). In all cases (SiR- or TAMRA-labeled GCE-tagged cellular structures), signal intensity levels were considerably higher than background levels measured in cells expressing a plasmid encoding only the orthogonal tRNA/tRNA-synthetase pair and labeled with the corresponding Tet-conjugated Fl-dyes (Fig. 6g). Together, these results validate labeling specificity and compatibility of the GCE-tag for organelle labeling.

Successful and specific labeling was also obtained for GCE-tagged extracellular protein EGFR and intracellular ESCRT-III protein CHMP4B as indicated by in-gel fluorescence and live-cell imaging (Additional file 1: Figure S5). For EGFR labeling, the GCE-tag was inserted between the signal peptide and the protein coding sequence (Additional file 1: Figure S5c, d), indicating that the GCE-tag is not exclusive for the N-terminal. Thus, besides its use in organelle labeling, the GCE-tag can be used for labeling intra- and extracellular proteins in live cells.

**Fig. 7:**
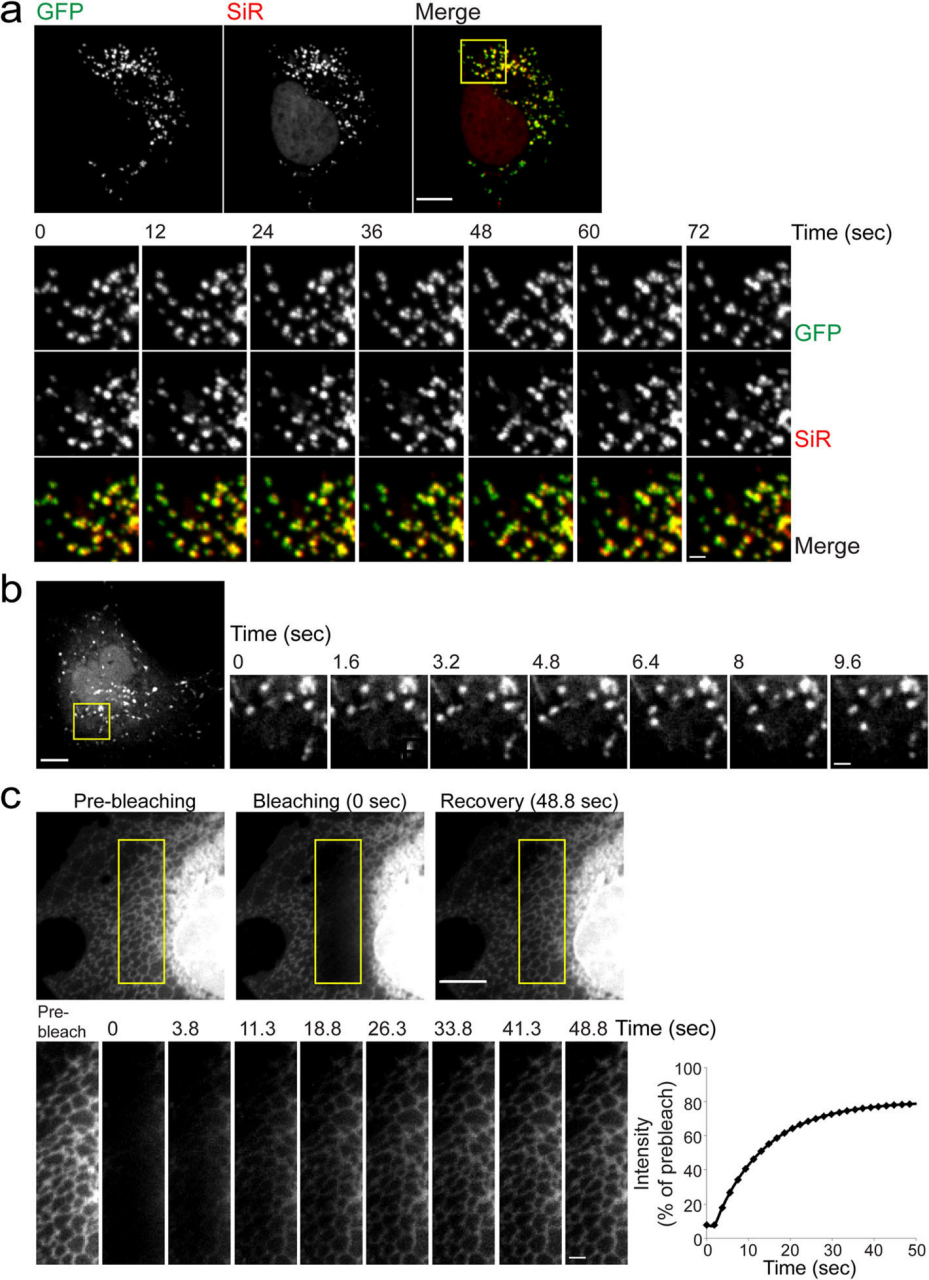
Live-cell recordings of cellular organelles labeled with GCE-tag and a tetrazine-conjugated Fl-dye. **a** Live-cell recordings of cells expressing GCE-tag-GFP-SKL labeled with SiR-Tet. Upper panel: maximum projection images of the entire cell taken at time 0. Bottom panel: sequential zoomed-in images of a subset of the cell (corresponds to square in upper panel) taken from the movie series. Every fourth frame from the movie series is shown (Additional file 4: Movie S3). **b** Live-cell recordings of cells expressing GCE-tag-CD63 labeled with TAMRA-Tet. Left, a maximum projection image of the entire cell taken at time 0. Sequential zoomed-in images of a subset of the cell (corresponds to square in left panel) taken from the movie series are shown to the right. Every fourth frame from the movie series is shown (Additional file 6: Movie S5). **c** FRAP analysis of cells expressing GCE-tag-ER^cb5^TM labeled with TAMRA-Tet. Snapshots of a subset of a representative cell taken from the movie series (Additional file 8: Movie S7) are shown in the upper panel. Sequential images of the bleached ROI are shown in the bottom panel (Additional file 9: Movie S8). Plot on the right represents the exponential fit of fluorescence intensity recovery versus time after photobleaching in the ROI. Intensity values were corrected for unintentional bleaching and normalized to intensity levels measured pre-bleaching. Results presented in each panel were obtained in at least 3 independent experiments. Scale-bars, 10 μm; zoomed-in images, 2 μm


Fl-dye labeling of the GCE-tagged constructs allowed live-cell recordings and tracking of any of the labeled cellular structures (i.e., MT, PM, peroxisomes, ER, MVB, and exosomes; Fig. 7 and Additional file 2: Movie S1, Additional file 3: Movie S2, Additional file 4: Movie S3, Additional file 5: Movie S4, Additional file 6: Movie S5, Additional file 7: Movie S6, Additional file 8: Movie S7, and Additional file 9 Movie S8). Fluorescence recovery after photobleaching (FRAP) experiments were successfully performed using the GCE-ER^cb5^TM ER marker, with comparable recovery times to those measured in the ER using VSVG-GFP [24] (Fig. 7c and Additional file 8: Movie S7, Additional file 9: Movie S8). It is worth mentioning that the overall size of the GCE-tag-ER^cb5^TM ER marker is considerably smaller than VSVG-GFP (GCE-tag-ER^cb5^TM, ~ 8.5 kDa; VSVG-GFP, ~ 84.5 kDa). Taken together, these results indicate that the newly developed GCE-based organelle markers reported here can be employed to study organelle dynamics in live cells with much smaller probes.

**Additional file 2:** **Movie S1.** Microtubule dynamics in cells labeled with GCE-tag-α-tubulin. COS7 cells expressing GCE-tag-α-tubulin and labeled with SiR-Tet were recorded at 4 s intervals. Shown are maximum intensity projections of 3 z-slices taken from a representative cell. Scale-bar: 10 μm.



**Additional file 4: Movie S3.** Peroxisome dynamics in cells labeled with GCE-tag-GFP-SKL. COS7 cells expressing GCE-tag-GFP-SKL and labeled with SiR-Tet were recorded at 5.3 s intervals. Left panel: 488 (GFP, green) channel, middle panel: 640 (SiR, red) channel. Shown are maximum intensity projections of 30 z-slices taken from a representative cell. Scale-bar: 10 μm.



**Additional file 8: Movie S7.** Visualizing ER dynamics by applying FRAP to GCE-tag-ER^cb5^TM labeled ER. COS7 cells expressing GCE-tag-ER^cb5^TM labeled with TAMTA-Tet were imaged for 2 min with 2 s intervals. Photobleaching was performed after 4 baseline timepoints (12 s). Shown are maximum intensity projections of 3 z-slices taken from a representative cell. Scale-bar: 10 μm.


**Fig. 8:**
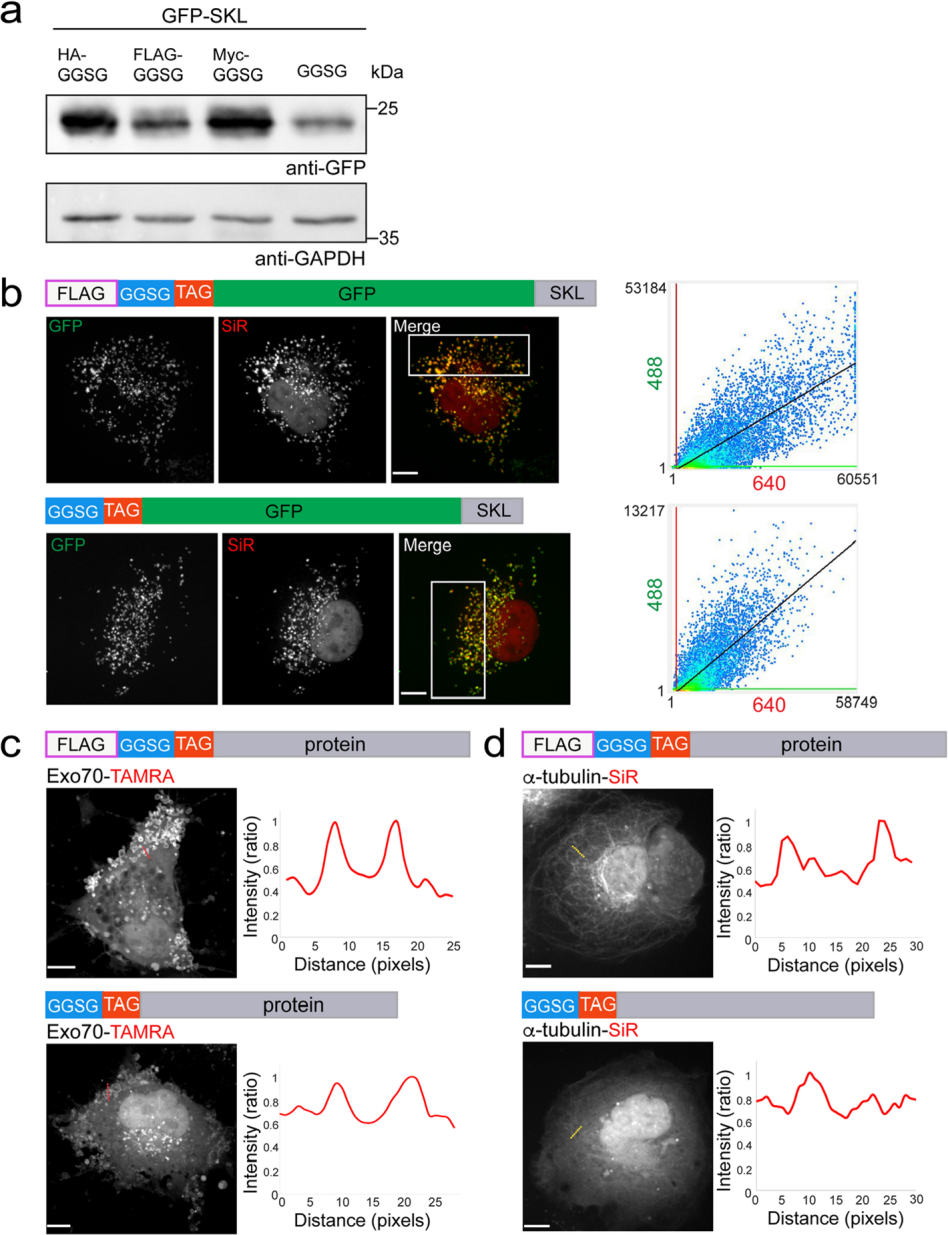
The GCE-tag is versatile and in some cases can be further minimized. **a** Western blot analysis of HEK293T cells transfected with pBUD-Pyl-RS plasmid inserted with GFP-SKL N-terminally tagged with (from left to right) the optimized GCE-tag (HA-GGSG-ncAA), tags with alternative short epitopes FLAG-GGSG-ncAA and Myc-GGSG-ncAA, and a tag with no epitope (GGSG-ncAA). Cells were incubated for 48 h in the presence of BCN-Lys, harvested and subjected to western blot analysis using anti-GFP antibodies. **b**–**d** Maximum intensity projections taken from live-cell images of COS7 cells transfected with FLAG-GGSG-ncAA (upper panels) or GGSG-ncAA (lower panels) conjugated to GFP-SKL (**b**), Exo70 (**c**), or α-tubulin (**d**) and labeled with the indicated tetrazine dyes. Plots in **b** represent co-localization analysis between intensity values of the 640 (SiR) and 488 (GFP) channels performed on large subsets of the cells that exclude the nucleus (correspond to rectangles in merged image); Pearson’s correlation values: upper panel, 0.87; bottom panel, 0.76. **c**, **d** Left, maximum intensity projection of representative cells. Right, line intensity profiles measured for the dashed lines in left panels. Results presented in each panel were obtained in at least 3 independent experiments. Scale bar 10 μm

The 14-residue long GCE-tag described here comprises the complete sequence of the HA tag. As such, the HA epitope can still be exploited for other applications, such as immunoblot, immunofluorescence, and immunoprecipitation (Figs. 2b and 5d). To expand the versatility of the tag, we tested whether the HA epitope can be replaced by FLAG (8 residues) or Myc (10 residues) epitopes (Fig. 8 and Additional file 1: Figure S6). GFP-SKL constructs carrying HA/FLAG/Myc GCE-tags were successfully expressed in cells, with SiR-Tet fluorescence co-localizing with GFP (Pearson’s correlation values: FLAG, 0.87; Myc, 0.67) (Fig. 8a, b upper panel, and Additional file 1: Figure S6a, b). Efficient labeling of exosomes was also obtained with the FLAG and Myc tag versions conjugated to Exo70, as indicated by live-cell imaging and in-gel TAMRA fluorescence (Fig. 8c upper panel and Additional file 1: Figure S6c, e). MT labeling, however, appeared to be compromised using FLAG/Myc GCE-tags, with very few cells exhibiting MT staining using the Myc GCE-tag (Fig. 8d upper panel and Additional file 1: Figure S6d). Reduced specific labeling was also observed using in-gel SiR fluorescence (Additional file 1: Figure S6f). However, while MT staining was more prominent using FLAG GCE-tag, higher labeling was observed for Myc GCE-tag using in-gel fluorescence. This data suggests that in some cases, the total level of labeled protein does not reflect its ability to label a specific cellular structure. Collectively, these results demonstrate that the system is versatile, in terms of the epitope used in the tag. But, while the HA version of the tag appears to be robust, labeling using the other epitope versions needs to be evaluated more carefully.

Last, we tested how removing the epitope sequence from the GCE-tag will affect labeling (Additional file 1: Figure S1i). In other words, is a sequence encoding GGSG linker followed by a TAG is sufficient for GCE-based bioorthogonal labeling of proteins? Almost no specific labeling was obtained upon tagging α-tubulin with a GCE-tag that lacks the HA sequence both by in-gel fluorescence and live-cell imaging (Fig. 8d bottom panel and Additional file 1: Figure S6f). For peroxisomes and exosomes, specific labeling was obtained, albeit at reduced levels (Fig. 8b, c bottom panels). In peroxisomes, the reduced labeling was consistent with the reduced expression of the protein observed by western blot (Fig. 8a, b bottom panel; Pearson’s correlations, 0.76). Reduced specific labeling was also observed for Exo70 using in-gel fluorescence (Additional file 1: Figure S6e), raising the possibility that the reduced labeling observed in cells is a result of failure to incorporate the ncAA during translation. Therefore, in specific cases in which the length of the tag is critical for preserving the function of the protein, the GCE-tag may be reduced to as few as five residues. Yet, this comes at the price of labeling efficiency and thus should be tested on a case-by-case basis.

## Discussion

In this work, we present a generic, small tag for labeling proteins in live mammalian cells with Fl-dyes through GCE and bioorthogonal chemistry, using a single expression vector. Efficient and specific labeling with Fl-dyes was observed for various intracellular structures and compartments, including MTs, PM, exosomes, lysosomes, MVBs, peroxisomes, and ER using appropriate tag-bearing markers, and for extracellular and cytosolic proteins that carry the tag. By adding 14 residues to the N-terminal of proteins, we minimized the need for prior knowledge on the protein and bypassed the screening step currently associated with the technique. Moreover, by inserting the TAG codon at the beginning of the coding sequence (rather than in the middle of the sequence), we avoided the expression of truncated proteins. As labeling efficiencies obtained using the GCE-tag were either comparable or superior to site-specific labeling, the GCE-tag presented here provide an attractive, easy-to-implement, alternative for bioorthogonal labeling of proteins modified to carry a ncAA.

The GCE-tag reported here is considerably smaller than Fl-protein tags and self-labeling protein tags (GCE-tag, ~ 1.5 kDa; GFP, 27 kDa; SNAP-tag, 19 kDa) [1, 2]. The only tag with a comparable size to that of the GCE-tag is the 12 amino-acid-long FlAsH tag [25]. However, labeling proteins with FlAsH tags rely on biarsenical-functionalized fluorescent dyes, which exhibit unspecific binding to cellular membranes and are toxic to cells [2]. Therefore, although adding a tag is not as elegant as site-specific labeling, the GCE-tag stands as one of the shortest tags available for fluorescence labeling of proteins and organelles in live cells.

Using GCE-based bioorthogonal labeling is known to suffer from relatively high noise levels compared to fluorescent protein tag-based approaches. The main noise sources are non-specific binding of the tetrazine-conjugated fluorescent dyes and excess of tRNA that is charged with a ncAA and is free to undergo the bioorthogonal reaction. The latter is the main source for the unspecific staining observed in the nucleolus. To minimize these noise factors, we previously performed careful optimization of all assay components [6, 19]. Using our optimized labeling assay conditions, GCE-tag-based labeling was comparable to protein tags in terms of labeling efficiencies and SNR values. Given the small size of the tag, which is likely to better preserve the physiological properties of proteins and organelles, we find GCE-tag superior to conventional protein tags for protein labeling.

While demonstrating the suitability of the GCE-tag for labeling proteins in a variety of cellular compartments, the results described here stress the need to test several dyes when calibrating conditions for bioorthogonal labeling. Table 1, summarizing the dyes found suitable for labeling each organelle, can be used as a guideline when optimizing labeling conditions for different cellular proteins. The increasing number of commercially available tetrazine-conjugated dyes will no doubt expand the ability to tailor Fl-dyes to specific cellular environments.

**Table 1:** List of organelle markers labeled via bioorthogonal labeling using the GCE-tag

Organelle	Marker name	Fl-dye-Tet	Notes
Microtubules	α-Tubulin	SiR	
Cell membrane	CAAX	SiR/TAMRA	GFP conjugated
Peroxisomes	SKL	SiR/TAMRA	GFP conjugated
Lysosomes	Lamp1	SiR	
ER	ER^cb5^TM	TAMRA	
Exosomes	Exo70	TAMRA	
MVB's	CD63	TAMRA	

Successful GCE-based bioorthogonal labeling mainly relies on two steps: expression of a protein carrying the ncAA, and a bioorthogonal reaction between the ncAA and the Fl-dye. We find that protein expression levels do not always correlate with efficient labeling. In some cases, lower expressions of the modified protein resulted in higher SNRs. When labeling cellular structures, such behavior can result from expressing the modified proteins at levels, which are higher than the capacity of the cellular structure. In such cases, the excess of overexpressed protein will remain in the cytosol, leading to increased background levels and consequently lower SNRs. Maintaining low expression levels of the modified protein is preferential for cell physiology. Therefore, upon applying GCE-based labeling, we recommend testing both parameters and choosing the lowest expression conditions that provide the highest SNRs.

The use of the GCE-tag reported here expands the plethora of labeling options for proteins in live cells. As with other labeling approaches, GCE-tag-based bioorthogonal labeling can be combined with conventional labeling approaches for multicolor labeling and can be tailored for super-resolution microscopy [26]. Moreover, the approach can potentially be expanded to labeling endogenous proteins using genome-editing techniques and can be further applied to recently reported GCE-modified tissues and organisms [27–31]. Additionally, a variety of ncAAs that carries different chemical functionalities has been genetically encoded in mammalian cells [8, 32–35]. The GCE-tag can be further used for incorporating these ncAAs, expanding its use in labeling applications and beyond.

## Materials and methods

### Cell culture

COS7 and HEK293T cells were kind gifts from Marcelo Ehrlich (Tel Aviv University). Cells were grown in Dulbecco’s modified Eagle’s medium (DMEM; Life Technologies, Carlsbad, CA) supplemented with 10% fetal bovine serum (FBS), 2 mM glutamine, 10,000 U/ml penicillin, and 10 mg/ml streptomycin.

### Plasmids and constructs

Tags were sub-cloned into the single expression vector pBUD-BCNK-RS that carries *pylT* encoding for tRNA_CUA_^Pyl^, and Pyrrolysyl-tRNA synthetase [18] (Additional file 1: Figure S1), using *NotI/KpnI* restriction sites. Sequences encoding organelle markers or proteins of interest were then inserted in-frame using the *KpnI/XhoI* restriction sites at pBUD-BCNK-RS. All constructs were sequenced before use.

### Incorporation of the ncAA to proteins in cells

Assay was performed according to our previously optimized protocol [6]. Twenty-four hours before transfection, cells were plated at 20% confluency using the following dishes: live cell imaging, 4-well chamber slide (Ibidi, Martinsried, Germany); western blot, 12-well plate (NUNC, Rochester, NY); and immunostaining, #1.0 coverslips (Menzel, Braunschweig, Germany). Cells were transfected with pBUD-BCNK-RS plasmids carrying different tags and organelle markers (Additional file 1: Figure S1, Table S1) using Lipofectamine 2000 (Life Technologies, Carlsbad, CA) according to the manufacturer’s protocol, and incubated for 48 h in the presence of the ncAA BCN-Lys (0.5 mM, Synaffix, Oss, Netherlands) in growth media supplemented with 100 μM ascorbic acid (Sigma Aldrich, Israel).

### Bioorthogonal labeling

Forty-eight hours post-transfection, cells were washed with fresh medium (3 × quick wash followed by 3 × 30 min wash) at 37 °C, incubated with SiR-Tet (1–2 μM, 1 h, Spirochrome, Stein am Rhein, Switzerland) or TAMRA-Tet (2 μM, 1 h, Jena BioScience, Germany), and washed again with fresh medium (3 × quick wash and 3 × 30 min wash) at 37 °C.

### In-gel fluorescence

Forty-eight hours post-transfection, cells were labeled with the appropriate Fl-dye as described above and in Table 1. After labeling, cells were collected, centrifuged at 200×*g* for 5 min, and washed with PBS twice. Then, cells were lysed using RIPA lysis buffer (150 mM NaCl, 1% NP-40, 0.5% deoxycholate, 0.1% SDS, 50 mM Tris [pH 8.0]) supplemented with complete protease inhibitor for 30 min at 4 °C. Total protein concentrations were measured with BCA Protein Assay Kit (Pierce Biotechnology), and equal total protein amounts were loaded on an SDS-PAGE gel. Gels were imaged at the appropriate wavelength using a Typhoon FLA 7000 biomolecular imager (GE Healthcare, PA, USA) to reveal specific Fl-dye labeling.

### Western blot

Cells were harvested 48 h post-transfection and lysed using RIPA lysis buffer supplemented with complete protease inhibitor for 30 min at 4 °C. Total protein concentrations were measured with BCA Protein Assay Kit, and equal total protein amounts were loaded and were subjected to western blot analysis using the following primary antibodies: rabbit anti-HA (1:4000, catalog number G166, Applied Biological Materials, Richmond, Canada, RRID, AB_2813867), mouse anti-GAPDH (1:1000, catalog number G041, Applied Biological Materials, RRID, AB_2813868), mouse anti-GFP (1:1000, catalog number G096, Applied Biological Materials, RRID, AB_2813869), and rabbit or mouse-peroxidase secondary antibodies (1:10,000, catalog number 715-035-151 or 711-035-152, Jackson ImmunoResearch, West Grove, PA, RRID, AB_2340771 or AB_10015282).

### Immunofluorescence

Cells were fixed 48 h post-transfection with 4% paraformaldehyde (PFA) and co-stained with rabbit anti-HA (1:500) and mouse anti-CD63 (1:200, catalog number ab59479 Abcam, Cambridge, MA, RRID, AB_940915) primary antibodies and with Alexa Fluor 488 anti-rabbit and Alexa Fluor 594 anti-mouse (1:500, catalog number A21206 or A21203 (RRID), Life Technologies; RRID: AB_2535792 or AB_2535789) secondary antibodies. Cells were mounted with Fluoromount-G (SouthernBiotech, Birmingham, AL).

### Live cell imaging

Cells were imaged on a fully incubated confocal spinning-disk microscope at 37 °C (Marianas; Intelligent Imaging, Denver, CO) using a × 63 oil objective (numerical aperture 1.4); depending on the protein expression levels of each cell, the specified Fl-dye or FP was excited at laser powers between 5 and 40% for 100–350 ms, and recorded on an electron-multiplying charge-coupled device camera (pixel size, 0.079 μm; Evolve; Photometrics, Tucson, AZ). A total of 3–30 confocal slices were captured for each image.

### Image processing

Analysis of image sets was performed using SlideBook, version 6 (Intelligent Imaging, Denver, CO). To improve visibility, some of the images were subjected to unsharp Mask, Gaussian filter, or both. Care was taken to apply similar filtering for all channels acquired in the same image and for all images that represent the same cellular structure, to allow for comparison. For line intensity analysis, the intensity levels along a line that crosses the cellular structure were measured. Intensity levels were normalized to 1 after background subtraction and plotted. SNRs for each cellular structure were calculated by dividing the maximum intensity by the minimum intensity obtained in each line intensity plot. Intensity levels shown in Fig. 6g were calculated by dividing the mean intensity value obtained inside the cell by the mean intensity value measured outside the cell. SNR and intensity measurements for each cellular structure were taken from at least ten different cells from three independent experiments. Plots presented in Fig. 4f, g were generated by segmenting puncta structures using an intensity mask in SlideBook, based on the GFP channel (f) or the SiR channel (g) and plotting the intensity in one channel against the intensity in the other channel. All values were subjected to background subtraction. Pearson’s correlation analysis was performed for the indicated regions of interest, using the co-localization plugin in SlideBook. Low intensity levels were filtered out to avoid noise measurements (indicated in green and red lines in plots). Analysis was performed after background subtraction.

### FRAP analysis

FRAP experiments were performed using the same confocal spinning-disk microscope setup, on cells expressing GCE-tag-ER^cb5^TM and labeled with TAMRA-Tet. After four baseline time points, bleaching was carried out on a selected ROI using 100 ms exposure to a 405 nm laser. Recovery after bleaching was recorded for 1 min with 1.8 s intervals. FRAP analysis fitting and unintentional bleaching corrections were performed using SlideBook.

### Statistical analysis

Statistical analysis was calculated in GraphPad Prism version 5.00 for Windows (La Jolla, CA, USA). Data are shown as means ± SEM. Statistical significance was determined by ANOVA or *t* test analysis: ***p < 0.0001, **p = 0.001–0.01, *p = 0.01–0.05, and ns *p* > 0.05.

## Supplementary information

**Additional file 1: Figure S1.** Single expression vector maps and sequences of the designed linkers. **Figure S2.** Unsuccessful attempt for MT labeling using Met-TAG-α-tubulin. **Figure S3.** Unsuccessful attempt of labeling of MVBs, Exosomes and ER using the GCE-tag and SiR-Tet labeling. **Figure S4.** Unsuccessful mitochondria labeling using the GCE-tag. **Figure S5.** Labeling cellular proteins using the GCE-tag. **Figure S6.** Evaluating peroxisomes, exosomes and MTs labeling using Flag/Myc- GGSG-TAG or GGSG-TAG as GCE-tags. **Table S1.** Sequences of organelle markers used in this work.

**Additional file 3: Movie S2.** Plasma membrane dynamics in cells labeled with GCE-tag-GFP-CAAX. COS7 cells expressing GCE-tag-GFP-CAAX were labeled with SiR-Tet and incubated in serum-free DMEM for 6 h. Cells were recorded at 5.5 s intervals. Left panel: 640 (SiR, red) channel, middle panel: 488 (GFP, green) channel, right panel: overly. Shown are maximum intensity projections of 3 z-slices taken from a representative cell. Scale-bar: 10 μm.

**Additional file 5: Movie S4.** Lysosome dynamics in cells labeled with GCE-tag-Lamp1 and treated with chloroquine. COS7 cells expressing GCE-tag-Lamp1 and labeled with SiR-Tet were imaged for 3 h in the presence of chloroquine (120 μM), at 10 min intervals. Shown are maximum intensity projections of 20 z-slices taken from a representative cell. Scale-bar: 10 μm.

**Additional file 6: Movie S5.** MVB dynamics in cells labeled with GCE-tag-CD63. COS7 cells expressing GCE-tag-CD63 and labeled with TAMRA-Tet were recorded at 0.4 s intervals. Shown are maximum intensity projections of 20 z-slices taken from a representative cell. Scale-bar: 10 μm.

**Additional file 7: Movie S6.** Exosome dynamics in cells expressing GCE-tag-Exo70. COS7 cells expressing GCE-tag-Exo70 and labeled with TAMRA-Tet were recorded at 1 s intervals. Single confocal slices taken from a representative movie are shown. Scale-bar: 10 μm.

**Additional file 9: Movie S8.** A Zoomed-in video of the bleached region in the ER. A Zoomed-in video of the bleached region shown in Additional file 8: Movie S7. Scale-bar: 2 μm.

## Data Availability

All data generated or analyzed during this study are included in this published article and its supplementary information files.
