# Supplementary material for: A straightforward approach for bioorthogonal labeling of proteins and organelles in live mammalian cells, using a short peptide tag
Source: BMC Biol. 2020 Jan 14;18:5. doi: 10.1186/s12915-019-0708-7 (PMC6961407; doi:10.1186/s12915-019-0708-7)
Supplement: Supplementary file 4 — Additional file 1: Figure S1. Single expression vector maps and sequences of the designed linkers. Figure S2. Unsuccessful attempt for MT labeling using Met-TAG-α-tubulin. Figure S3. Unsuccessful attempt of labeling of MVBs, Exosomes and ER using the GCE-tag and SiR-Tet labeling. Figure S4. Unsuccessful mitochondria labeling using the GCE-tag. Figure S5. Labeling cellular proteins using the GCE-tag. Figure S6. Evaluating peroxisomes, exosomes and MTs labeling using Flag/Myc- GGSG-TAG or GGSG-TAG as GCE-tags. Table S1. Sequences of organelle markers used in this work. [file 12915_2019_708_MOESM1_ESM.pdf]

Supplemental Information

A straightforward approach for bioorthogonal labeling of proteins and organelles in live mammalian cells, using a short peptide tag

Inbar Segal[1,2], Dikla Nachmias[1,2], Andres Konig[1,2], Ariel Alon[1,2], Eyal Arbely[2,3] and Natalie Elia[1,2]

[1] Department of Life Sciences, Ben-Gurion University of the Negev, Beer Sheva 84105, Israel

[2] National Institute for Biotechnology in the Negev (NIBN), Ben-Gurion University of the Negev, Beer Sheva 84105, Israel

[3] Department of Chemistry, Ben-Gurion University of the Negev, Beer Sheva 84105, Israel

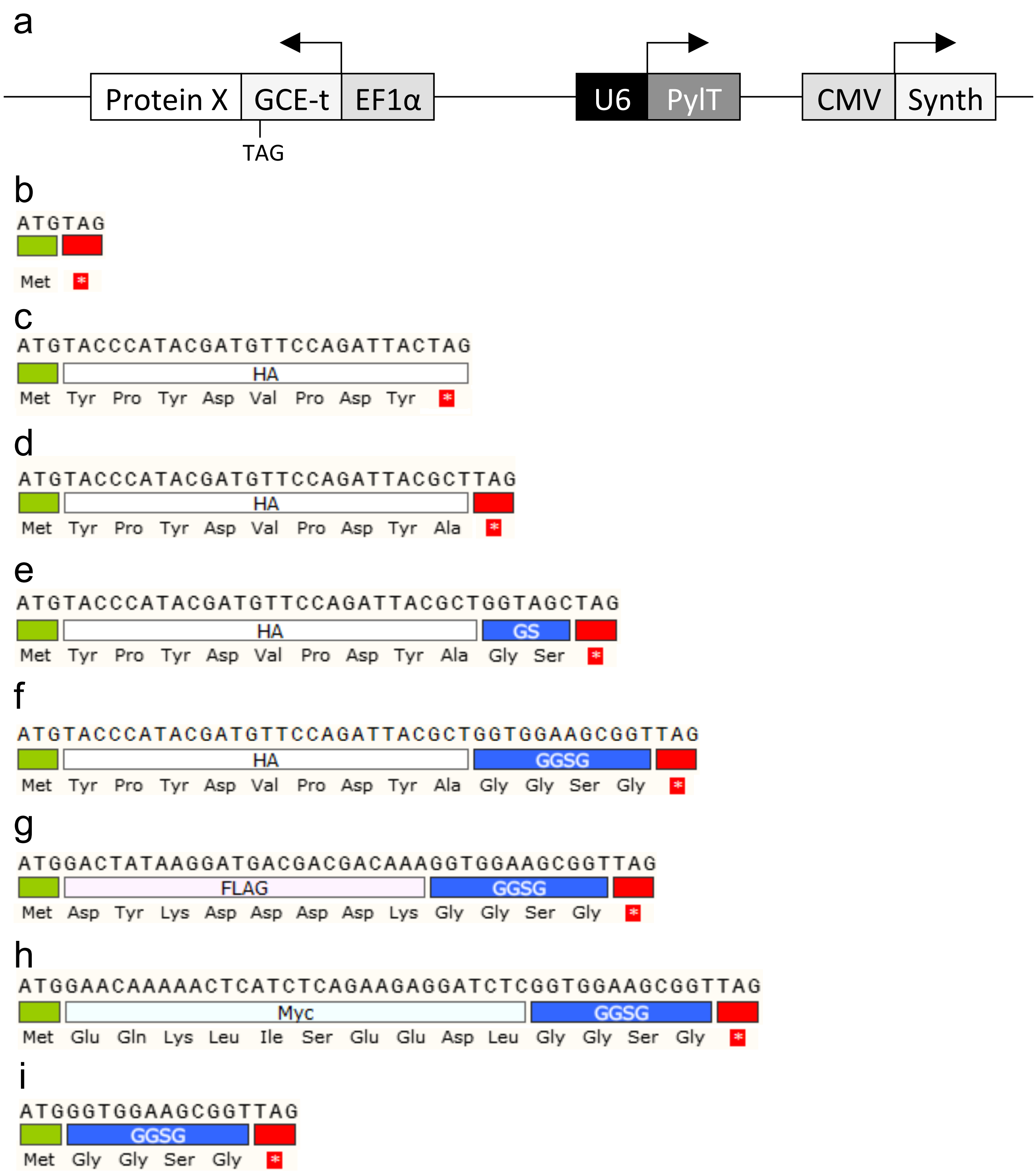

Figure S1. Single expression vector maps and sequences of the designed linkers

(a) Schematic representation of the genetic code expansion vector with *PylT* gene and BCN-RS. (b-i) Nucleotide sequences of (b) Methionine-TAG, (c) HA*-TAG (corresponds to tag 1 in Fig. 2a), (d) HA-TAG (corresponds to tag 2 in Fig. 2a), (e) HA-GS-TAG (corresponds to tag 3 in Fig. 2a), (f) HA-GGSG-TAG (corresponds to tag 4 in Fig. 2a), (g) Flag-GGSG-TAG, (h) Myc-GGSG-TAG, (i) GGSG-TAG. ATG represents beginning of ORF, ncAA incorporation site is at the TAG codon marked in red.

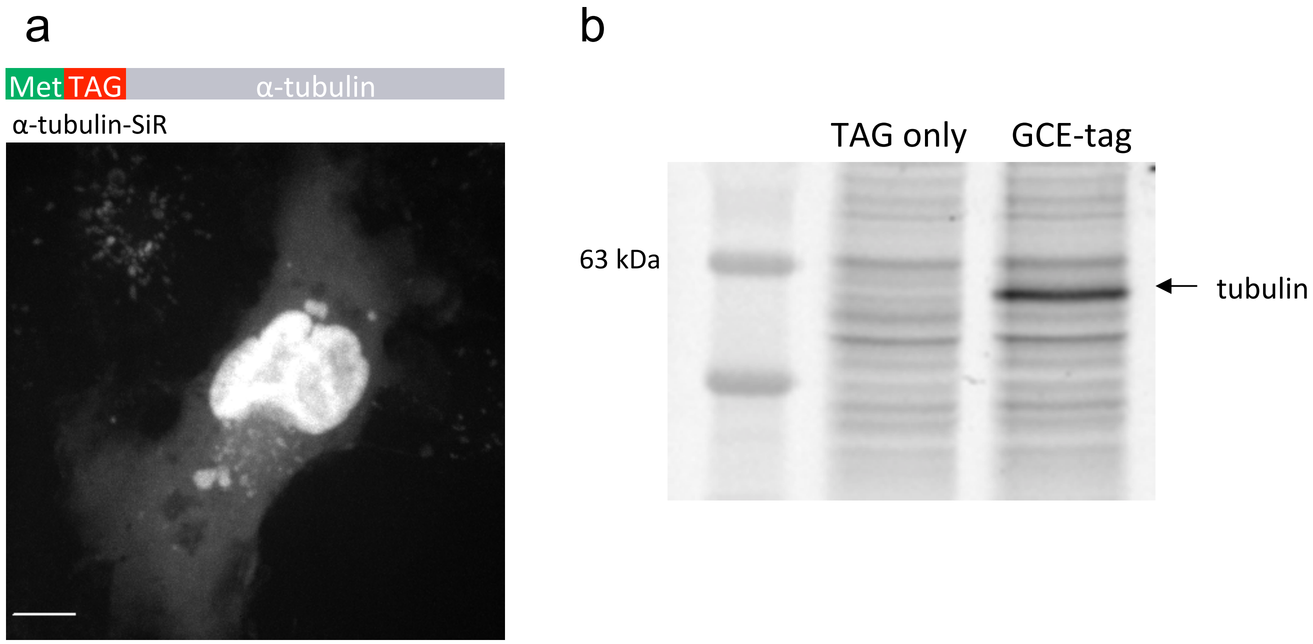

Figure S2. Unsuccessful attempt for MT labeling using Met-TAG-α-tubulin

COS7 (a) and HEK293T (b) cells were transfected with pBUD-Pyl-RS that carries Methionine-TAG-α-tubulin and labeled with SiR-Tet as described in the materials and methods section . Next, cells were imaged live (a) or lysed and subjected to in-gel fluorescence (b), as described in materials and methods. GCE-tag-α-tubulin is shown in (b) for comparison. Image in (a) is a maximum intensity projection of 30 z-slices taken from a representative cell. Note that there no MT labeling and no specific labeling of α-tubulin is obtained using this tag. Scale-bar: 10 μm

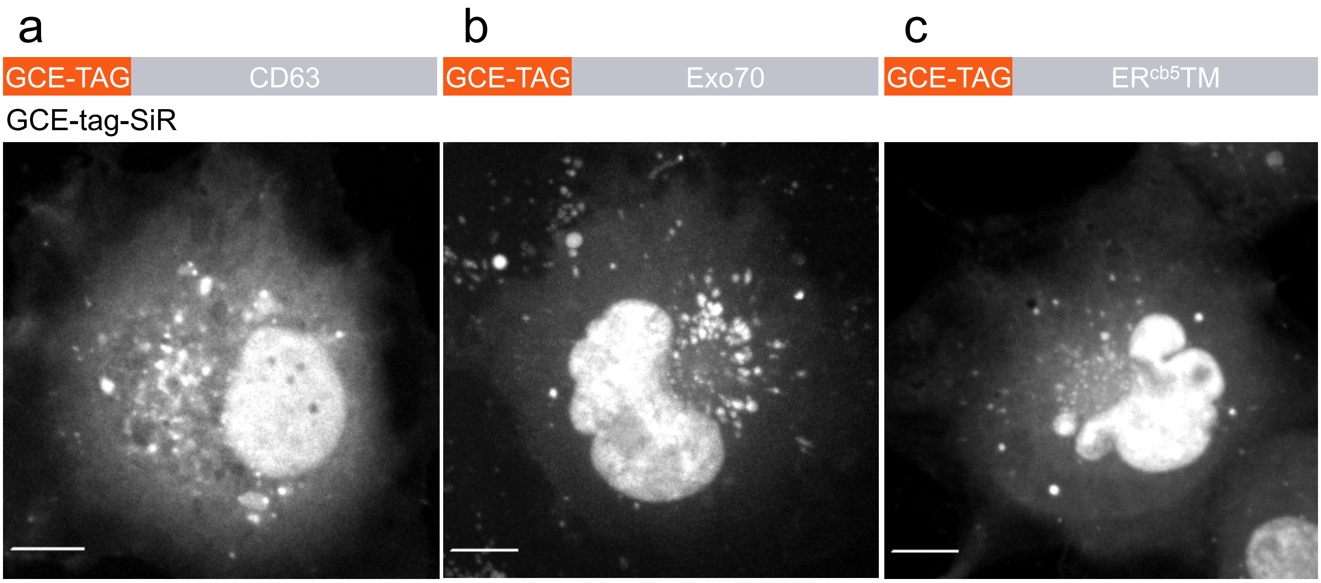

Figure S3. Unsuccessful attempt of labeling of MVBs, Exosomes and ER using the GCE-tag and SiR-Tet labeling

Maximum intensity projections taken from live cell images of COS7 cells transfected with pBUD-Pyl-RS carrying GCE-tag-CD63 (a), GCE-tag-Exo70 (b) or GCE-tag-ER^{cb5}TM (c) and labeled with SiR-Tet. No specific labeling was obtained under these conditions. Scale-bar: 10 µm.

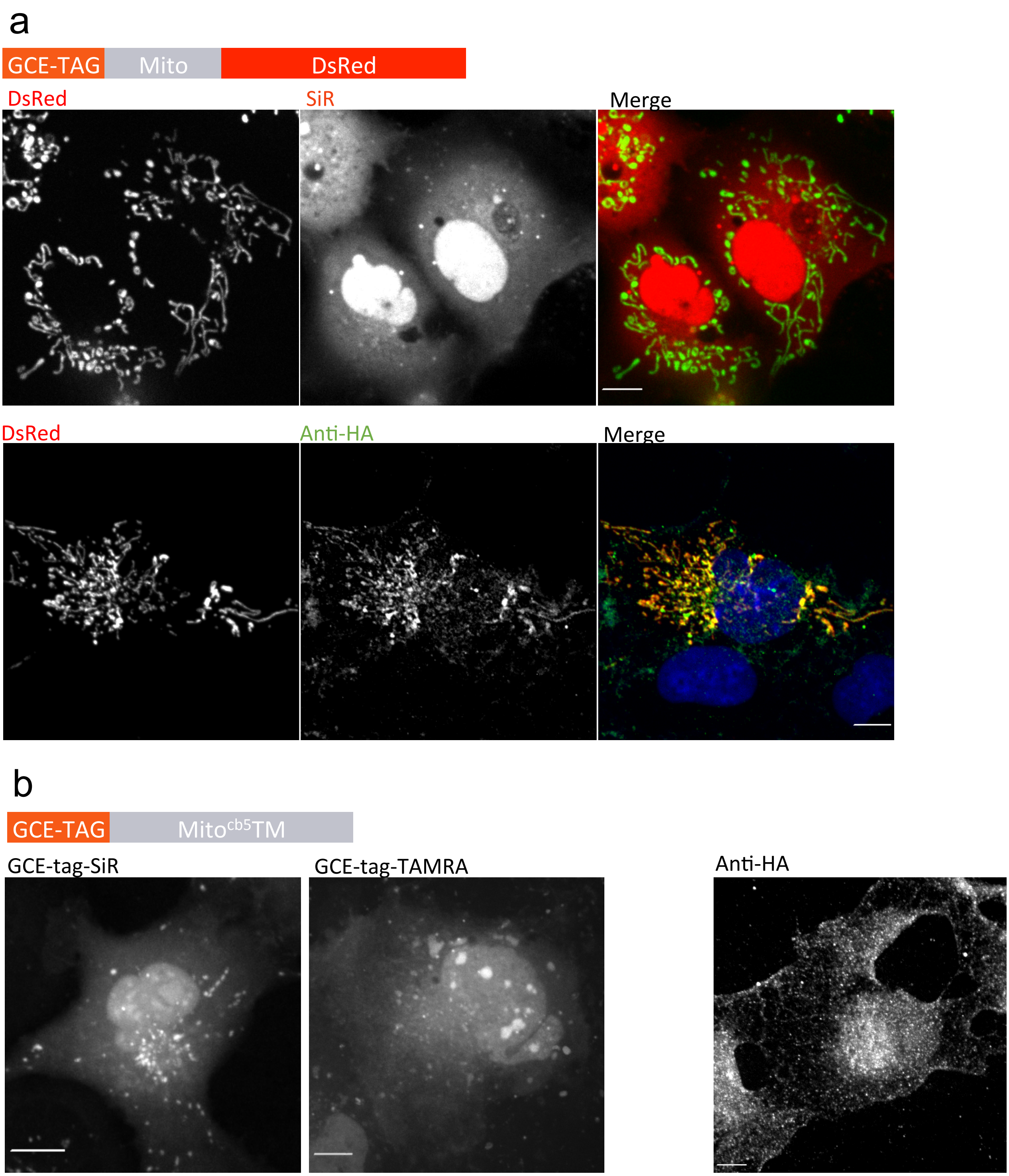

Figure S4. Unsuccessful mitochondria labeling using the GCE-tag

COS7 cells were transfected with pBUD-Pyl-RS carrying GCE-tag-MitoDsRed **(a)** or GCE-tag-ER^{cb5}TM **(b).** Cells were either labeled with TAMRA-Tet or SiR-Tet (as indicated) and imaged live (a top panel, b left and middle panels) or fixed and immunostained using anti-HA antibodies (a bottom panel, b right panel. In live cells, no specific labeling with Fl-dyes was obtained under any of these conditions. Scale-bar: 10 µm.

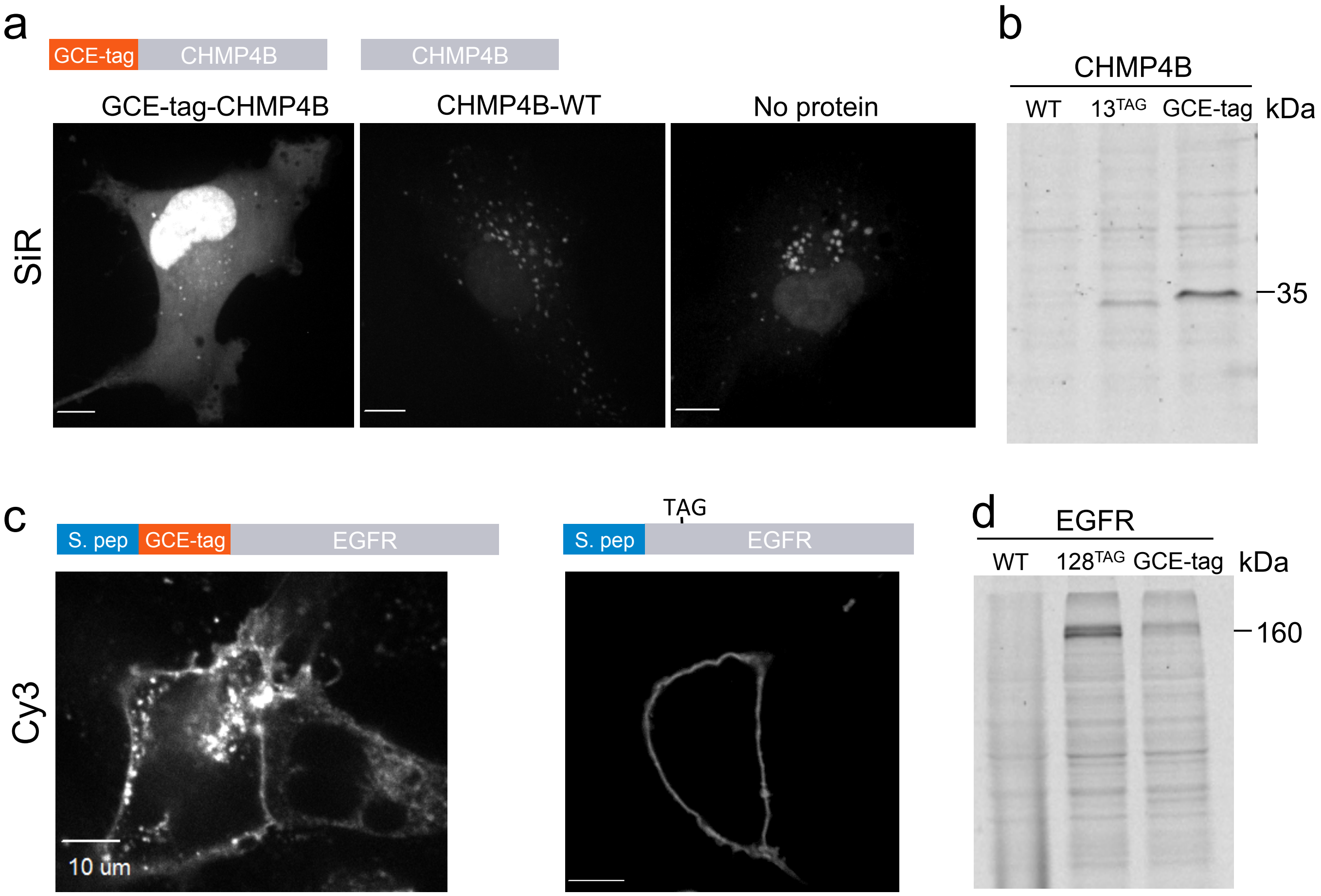

Figure S5. Labeling cellular proteins using the GCE-tag

(a,c) Maximum intensity projections taken from live cell images of COS7 cells, transfected with pBUD-Pyl-RS plasmid carrying the indicating constructs and labeled with with SiR-Tet (a) or with Cy3-Tet (c). **(b,d)** In-gel fluorescence analysis of HEK293T cells transfected as in (a or c, respectively) and labeled with SiR-Tet. **(a)** Maximum intensity projections. **(c)** Confocal slices. Position 13 in CHMP4B and 128 in EGFR were optimized for site specific labeling. Cy3-Tet is a cell impermeable dye. Intracellular labeling observed in (c) results from EGFR endocytosis. S.pep, signal peptide. Specific labeling was obtained for both CHMP4B and EGFR. Scale-bar: 10 μm.

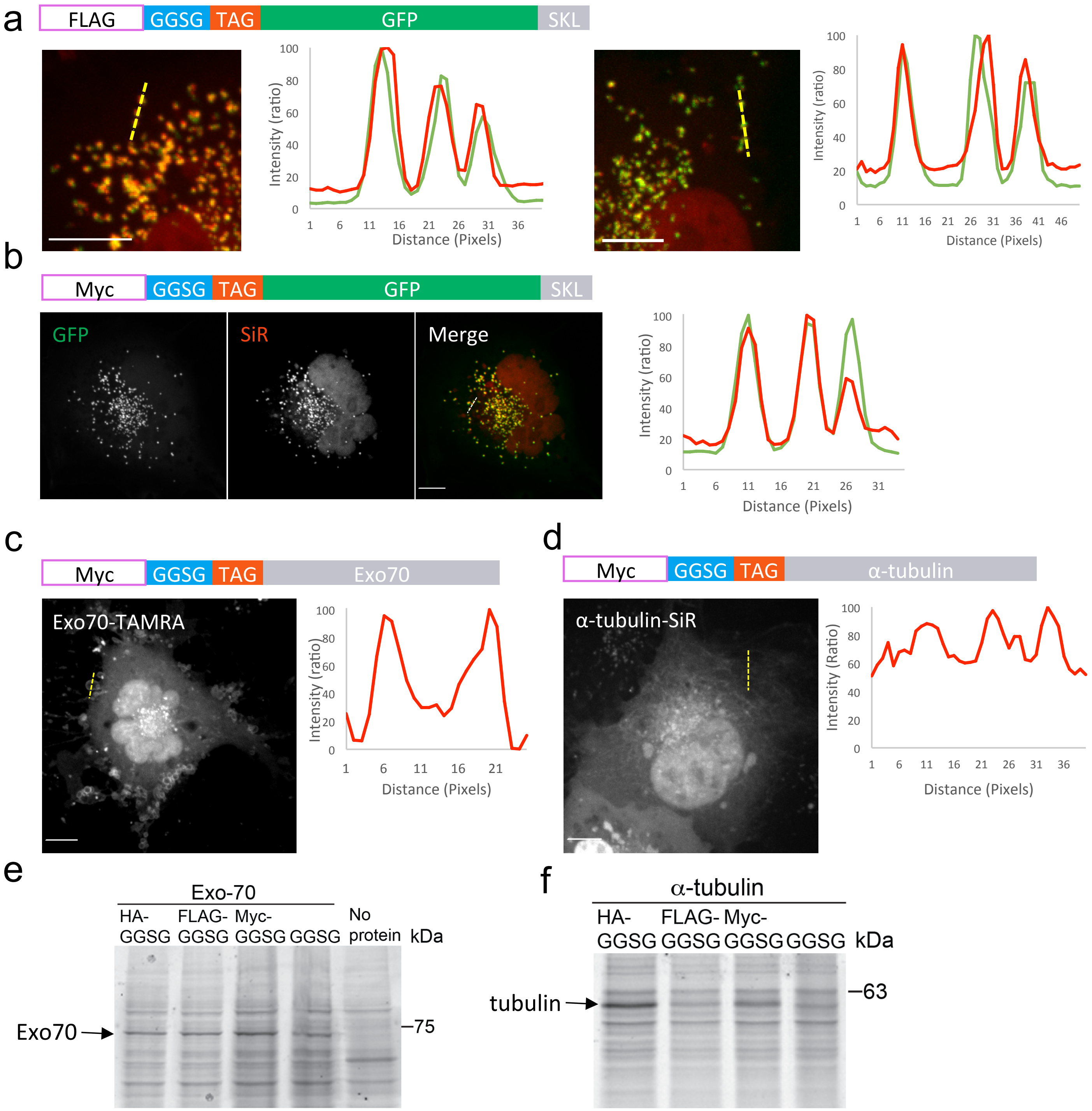

Figure S6. Evaluating peroxisomes, exosomes and MTs labeling using Flag/Myc-GGSG-TAG or GGSG-TAG as GCE-tags

(a) Zoomed-in images (maximum projections) of COS7 cells expressing FLAG-GGSG-TAG-GFP-SKL (left) or GGSG-TAG-GFP-SKL (right) and labeled with SiR (relates to Fig. 8b). Intensity profiles measured for the dashed line in each image are plotted to the right (GFP, green, SiR, red). **(b-d)** Maximum intensity projections taken from live cell images of COS7 cells, transfected with pBUD-Pyl-RS expressing Myc-GGSG-ncAA conjugated to: GFP-SKL **(b)**, Exo70 **(c)** or α-tubulin **(d)** and labeled with the indicated tetrazine dyes. Intensity profiles measured for the dashed line in each image are plotted to the right. **(e-f)** In-gel fluorescence analysis of HEK293T cells transfected as in (a-c) with the indicated tags and labeled with either TAMRA-Tet **(e)** or SiR-Tet **(f)**. Results were obtained in at least 3 independent experiments. Scale-bar: 10 µm.

Name	Sequence
**FRB-CAAX**	ADKQKNGIKVNFKIRHNIEDGSVQLADHYQQNTPIGDGPVLLPDNHYLSTQSKLSKDPNEKRDHMVLLE<br>FVTAAGITLGMDELYKSGLRSRAEMWHEGLEEASRLYFGERNVKGMFEVLEPLHAMMERGPQTLKETS<br>FNQAYGRDLMEAQEWCRKYMKSGNVKDLTQAWDLYYHVFRRISKQRNSAVDSGLRSKLNPPDESGPG<br>CMSCKCVLS
**Lamp1**	AAPGARRPLLLLLLAGLAHSAPALFEVKDNNGTACIMASFSASFLTTYDAGHVSKVSNMTLPASAEVLKN<br>SSSCGEKNASEPTLAITFGEGYLLKLTFTKNTTRYSVQHMYFTYNLSDTQFFPNASSKGPDTVDSTTDIK<br>ADINKTYRCVSDIRVYMKNVTIVLWDATIQAYLPSSNFSKEETRCPQDQPSPTTGPPSPSPPLVPTNPSV<br>SKYNVTGDNGTCLLASMALQLNITYMKKDNTTVTRAFNINPSDKYSGTCGAQLVTLKVGNKSRVLELQF<br>GMNATSSLFFLQGVQLNMTLPDAIEPTFSTSNYSLKALQASVGNSYKCNSEEHIFVSKALALNVFSVQVQ<br>AFRVESDRFGSVEECVQDGNNMLIPIAVGGALAGLVLIVLIAYLIGRKRSHAGYQTI
**CD63**	AVEGGMKCVKFLLYVLLLAFCACAVGLIAVGVGAQLVLSQTIIQGATPGSLLPVVIIAVGVFLFLVAFVGCC<br>GACKENYCLMITFAIFLSLIMLVEVAAAIAGYVFRDKVMSEFNNNFRQQMENYPKNNHTASILDRMQADF<br>KCCGAANYTDWEKIPSMSKNRVPDSCCINVTVGCGINFNEKAIHKEGCVEKIGGWLRKNVLVVAAAALGI<br>AFVEVLGIVFACCLVKSIRSGYEVM
**ER^{cb5}TM**	ESGGGSGGGGSGGGGSQSDKDVKYYTLEEIKKHNHSKSTWLILHHKVYDLTKFLEEHP
**Exo70**	RRREIEDKLKQEEETLSFIRDSLEKSDQLTKNMVSILSSFESRLMKLENSIIPVHKQTENLQRLQENVEKT<br>LSCLDHVISYYHVASDTEKIIREGPTGRLEEYLGSMAKIQKAVEYFQDNSPDSPELNKVKLLFERGKESLE<br>SEFRSLMTRHSKVISPVLVLDLISADDELEVQEDVVLEHLPESVLQDVIRISRWLVEYGRNQDFMNVYYQI<br>RSSQLDRSIKGLKEHFRKSSSSSGVPYSPAIPNKRKDTPTKKPIKRPGRDDMLDVETDAYIHCVSAFVRL<br>AQSEYQLLMGIIPEHHQKKTFDSLIQDALDGLMLEGENIVSAARKAIIRHDFSTVLTVFPILRHLKQTKPEF<br>DQVLQGTAASTKNKLPGLITSMETIGAKALEDFADNIKNDPDKEYNMPKDGTVHELTSNAILFLQQLLDFQ<br>ETAGAMLASQETSSSATSYNSEFSKRLLSTYICKVLGNLQLNLLSKSKVYEDPALSAIFLHNNYNYILKSLE<br>KSELIQLVAVTQKTAERSYREHIEQQIQTYQRSWLKVTDYIAEKNLPVFQPGVKLRDKERQMIKERFKGF<br>NDGLEELCKIQKAWAIPDTEQRDKIRQAQKSIVKETYGAFLHRYSSVPFTKNPEKYIKYRVEQVGDMIDR<br>LFDTSA
**EGFR**	RPSGTAGAALLALLAALCPASRALYPYDVPDYAEEKKVCQGTSNKLTQLGTFEDHFLSLQRMFNNCEVV<br>LGNLEITYVQRNYDLSFLKTIQEVAGYVLIALNTVERIPLENLQIIRGNMYYENSYALAVLSNYDANKTGLK<br>ELPMRNLQEILHGAVRFSNNPALCNVESIQWRDIVSSDFLSNMSMDFQNHLGSCQKCDPSCPNGSCW<br>GAGEENCQKLTKIICAQQCSGRCRGKSPSDCCHNQCAAGCTGPRESDCLVCRKFRDEATCKDTCPPL<br>MLYNPTTYQMDVNPEGKYSFGATCVKKCPRNYVVTDHGSCVRACGADSYEMEEDGVRKCKKCEGPC<br>RKVCNGIGIGEFKDSLSINATNIKHFKNCTSISGDLHILPVAFRGDSFTHTPPLDPQELDILKTVKEITGFLLI<br>QAWPENRTDLHAFENLEIIRGRTKQHGQFSLAVVSLNITSLGLRSLKEISDGDVIISGNKNLCYANTINWK<br>KLFGTSGQKTKIISNRGENSCKATGQVCHALCSPEGCWGPEPRDCVSCRNVSRGRECVDKCNLLEGE<br>PREFVENSECIQCHPECLPQAMNITCTGRGPDNCIQCAHYIDGPHCVKTCPAGVMGENNTLVWKYADA<br>GHVCHLCHPNCTYGCTGPGLEGCPTNGPKIPSIATGMVGALLLLLVVALGIGLFMRRRHIVRKRTLRRLL<br>QERELVEPLTPSGEAPNQALLRILKETEFKKIKVLGSGAFGTVYKGLWIPEGEKVKIPVAIKELREATSPKA<br>NKEILDEAYVMASVDNPHVCRLLGICLTSTVQLITQLMPFGCLLDYVREHKDNIGSQYLLNWCVQIAKGM<br>NYLEDRRLVHRDLAARNVLVKTPQHVKITDFGLAKLLGAEEKEYHAEGGKVPIKWMALESILHRIYTHQS<br>DVWSYGVTVWELMTFGSKPYDGIPASEISSILEKGERLPQPPICTIDVYMIMVKCWMIDADSRPKFRELII<br>EFSKMARDPQRYLVIQGDERMHLPSPTDSNFYRALMDEEDMDDVVDADEYLIPQQGFFSSPSTSRTPL<br>LSSLSATSNNSTVACIDRNGLQSCPIKEDSFLQRYSSDPTGALTEDSIDDTFLPVPEYINQSVPKRPAGSV<br>QNPVYHNQPLNPAPSRDPHYQDPHSTAVGNPEYLNTVQPTCVNSTFDSPAHWAQKGSHQISLDNPDY<br>QQDFFPKEAKPNGIFKGSTAENAEYLRVAPQSSEFIGA
**CHMP4B**	SVFGKLFGAGGGKAGKGGPTPQEAIQRLRDTEEMLSKKQEFLEKKIEQELTAAKKHGTKNKRAALQALK<br>RKKRYEKQLAQIDGTLSTIEFQREALENANTNTEVLKNMGYAAKAMKAAHDNMDIDKVDELMQDIADQQ<br>ELAEEISTAISKPVGFGEEFDEDELMAELEELEQEELDKNLLEISGPETVPLPNVPSIALPSKPAKKKEEED<br>DDMKELENWAGSM

Table S1. Sequences of organelle markers used in this work
